# Identification of transcriptional regulatory variants in pig duodenum, liver, and muscle tissues

**DOI:** 10.1093/gigascience/giad042

**Published:** 2023-06-24

**Authors:** Daniel Crespo-Piazuelo, Hervé Acloque, Olga González-Rodríguez, Mayrone Mongellaz, Marie-José Mercat, Marco C A M Bink, Abe E Huisman, Yuliaxis Ramayo-Caldas, Juan Pablo Sánchez, Maria Ballester

**Affiliations:** Animal Breeding and Genetics Program, IRTA, Torre Marimon, Caldes de Montbui (08140), Spain; GABI, Université Paris-Saclay, INRAE, AgroParisTech, Jouy-en-Josas (78350), France; Animal Breeding and Genetics Program, IRTA, Torre Marimon, Caldes de Montbui (08140), Spain; GABI, Université Paris-Saclay, INRAE, AgroParisTech, Jouy-en-Josas (78350), France; IFIP-Institut du porc and Alliance R&D, Le Rheu (35651), France; Hendrix Genetics Research Technology & Services B.V., Boxmeer (5830 AC), The Netherlands; Hypor B.V., Boxmeer (5830 AC), The Netherlands; Animal Breeding and Genetics Program, IRTA, Torre Marimon, Caldes de Montbui (08140), Spain; Animal Breeding and Genetics Program, IRTA, Torre Marimon, Caldes de Montbui (08140), Spain; Animal Breeding and Genetics Program, IRTA, Torre Marimon, Caldes de Montbui (08140), Spain

**Keywords:** eQTL, hotspot, pig, RNA-Seq, WGS

## Abstract

**Background:**

In humans and livestock species, genome-wide association studies (GWAS) have been applied to study the association between variants distributed across the genome and a phenotype of interest. To discover genetic polymorphisms affecting the duodenum, liver, and muscle transcriptomes of 300 pigs from 3 different breeds (Duroc, Landrace, and Large White), we performed expression GWAS between 25,315,878 polymorphisms and the expression of 13,891 genes in duodenum, 12,748 genes in liver, and 11,617 genes in muscle.

**Results:**

More than 9.68 × 10^11^ association tests were performed, yielding 14,096,080 significantly associated variants, which were grouped in 26,414 expression quantitative trait locus (eQTL) regions. Over 56% of the variants were within 1 Mb of their associated gene. In addition to the 100-kb region upstream of the transcription start site, we identified the importance of the 100-kb region downstream of the 3′UTR for gene regulation, as most of the *cis*-regulatory variants were located within these 2 regions. We also observed 39,874 hotspot regulatory polymorphisms associated with the expression of 10 or more genes that could modify the protein structure or the expression of a regulator gene. In addition, 2 motifs (5′-GATCCNGYGTTGCYG-3′ and a poly(A) sequence) were enriched across the 3 tissues within the neighboring sequences of the most significant single-nucleotide polymorphisms in each *cis*-eQTL region.

**Conclusions:**

The 14 million significant associations obtained in this study are publicly available and have enabled the identification of expression-associated *cis*-, *trans*-, and hotspot regulatory variants within and across tissues, thus shedding light on the molecular mechanisms of regulatory variations that shape end-trait phenotypes.

## Background

Over the past decade, genome-wide association studies (GWAS) have been applied to study the association between genetic variants distributed across the genome of a species and traits of interest. Whether these traits are related to disease in humans or to production or health in livestock species, GWAS have shown that more than 88% of phenotype-associated variants are located outside protein-coding regions and are enriched in gene regulatory regions [1, 2]. These noncoding variants may affect traits of interest by acting on gene regulation mechanisms, for example, by affecting gene expression [3]. In this sense, gene expression can be considered an “intermediate phenotype,” as it is expected to be more closely linked to genetic variations than conventional phenotypes [4]. Genetic variants that are significantly associated with the expression of a gene are called expression quantitative trait loci (eQTLs), and they are commonly identified through expression GWAS (eGWAS), in which the expression of each gene is considered a trait to pinpoint genomic regions involved in its regulation. If the distance of an eQTL relative to its associated gene is less than 1 Mb, it is usually classified as a *cis*-eQTL [5]. Conversely, eQTLs located farther than 1 Mb from their associated genes or on another chromosome are referred to as *trans*-eQTLs.

In pigs, previous eQTL studies have been performed in a breed-specific context using either low-density genotyping arrays or gene expression arrays [6–9]. Nowadays, eGWAS can be conducted with the comprehensive set of polymorphisms that segregate in a population thanks to the use of whole-genome sequencing (WGS) data and genotype imputation. Such a strategy has the potential to reveal causal mutations responsible for the variation in gene expression levels. In humans, eGWAS have helped to detect the causal mutation for rare diseases, but only few studies have specifically aimed to uncover causal mutations associated with the transcriptomic variation of more than a particular set of genes [10]. Whole-genome datasets are composed of millions of variants, thus increasing their complexity and the computing power required to analyze them. Furthermore, transcriptome datasets can include measures for tens of thousands of genes, further complicating the interpretation of results. For this reason, the majority of studies using both types of datasets have focused on identifying *cis*-eQTL regions associated with genes of interest, disregarding or analyzing to a lesser extent *trans*-eQTL regions due to their complexity, small effect size, demanding computing requirements, and indirect regulation mechanisms [4, 5, 11, 12]. Regions upstream from the transcription start site (TSS) have received particular attention due to their potential role as promoter or enhancer regions. Nevertheless, other downstream regulatory regions should not be ignored.

Apart from human and mouse, few studies have analyzed how regulatory elements impact the phenotypes of other species [13]. To provide insight into the regulation mechanisms of gene expression in multiple tissues of livestock species, consortia such as FAANG (Functional Annotation of ANimal Genomes) and FarmGTEx (Farm Animal Genotype-Tissue Expression) were recently established [14, 15]. In this context, the GENE-SWitCH project [16] aims at characterizing the functional elements of chicken and pig genomes and describing genetic and epigenetic determinants of complex traits. Altogether, the functional annotation of genomes is expected to help advance genomic selection for the breeding industry toward more sustainable production systems. In addition, the identification of the regulatory regions responsible for the changes in gene expression may be translatable to other species. For example, pig biomedical models have played an important role for studying human diseases not only due to the similarity between both species in anatomical structure, genome, immunology, and physiology [17, 18] but also due to their similar gene expression profiles [19–21].

Our study was developed in the framework of the GENE-SWitCH project with the objective of discovering genetic polymorphisms associated with the variation of gene expression levels in the duodenum, liver, and muscle of pigs. In particular, polymorphisms were studied regarding the proximity to their associated gene, as well as their potential role as hotspot regions or causal mutations.

## Results

### Whole-genome and RNA sequencing

In this work, we sequenced the whole genome of 300 pigs from 3 commercial pig populations (Duroc, Landrace, and Large White), which resulted in 44,127,400 genetic variants. After the filtering steps on minor allele frequency and percentage missing, 25,315,878 polymorphisms remained for the association analyses. Variants were primarily classified as single-nucleotide polymorphisms (SNPs) (74.9%) and, to a lesser extent, insertions (13.9%) or deletions (11.2%). In addition, the transition/transversion ratio was 2.01.

**Figure 1:**
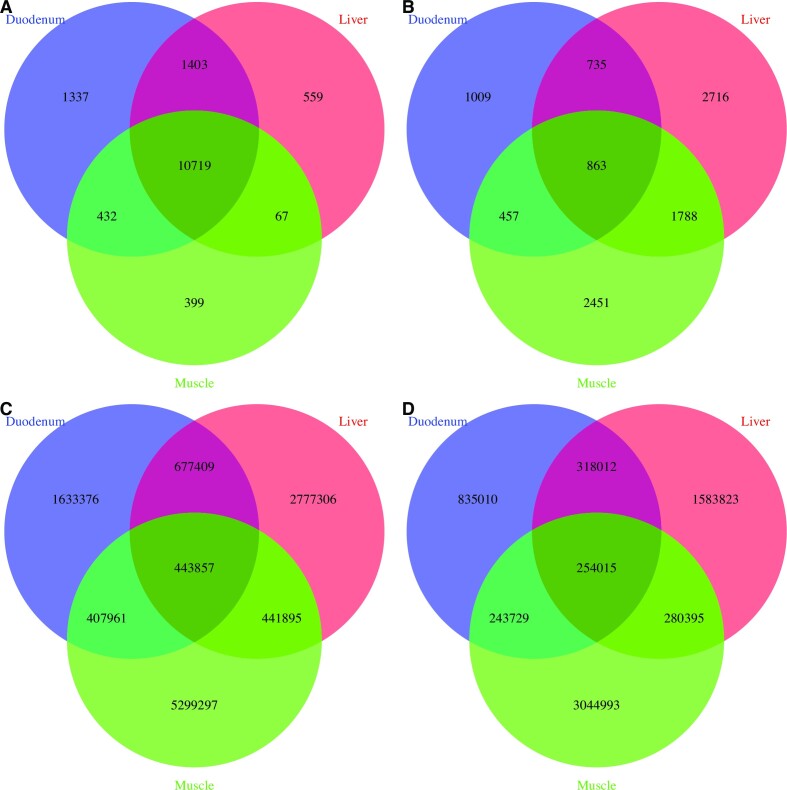
Venn diagram representing the shared elements across duodenum, liver, and muscle. (A) Number of expressed genes. (B) Number of expressed genes with at least 1 significantly associated variant. (C) Number of significantly associated variants. (D) Number of *cis*-regulatory variants.

In parallel, the transcriptomes of duodenum, liver, and skeletal muscle of the 300 pigs were sequenced. From the total sequenced reads, 92.1% were mapped against the pig reference genome. Out of these, 93.3% of the sequences were located inside gene regions (80.0% in exonic regions and 13.3% in intronic regions). After normalization, filtering, and quality control of the 3 transcriptomic datasets, 13,891 genes were found to be expressed in duodenum, 12,748 in liver, and 11,617 in muscle. The number of genes that were expressed in all 3 tissues was 10,719, while 14,916 genes were expressed in total among the 3 tissues (Fig. 1A).

### eGWAS

Among the 3 tissues, the eGWAS reported 14,096,080 significant associations (adjusted *P* ≤ 0.05) with 10,019 different genes (Table 1). The full list of significant associations found across tissues can be accessed in the “Availability of Data and Materials” section. The numbers of expressed genes that had at least 1 significantly associated variant were 3,064 in duodenum (22.1%), 6,102 in liver (47.9%), and 5,559 in muscle (47.9%) (Fig. 1B). Out of these genes with any significant association, 863 were expressed simultaneously in the 3 tissues. Regarding the number of associated variants shared between tissues, 3.1% of the variants were associated with the same gene in duodenum, liver, and muscle (Fig. 1C). Muscle was the tissue with the greatest number of unique associations, but duodenum and liver shared the greatest number of associated variants. Similar results were observed for the number of *cis*-regulatory variants shared across tissues, where 3.2% of the *cis*-regulatory variants were found in common (Fig. 1D).

**Table 1:** Number of significantly associated variants and eQTL regions per tissue

	Significantly associated variants		eQTL regions*	
Tissue	No.	*cis*-regulatory variants, No. (%^†^)	No.	*cis*-eQTL regions, No. (%^†^)
Duodenum	3,162,603	1,650,766 (52.2)	4,645	1,311 (28.2)
Liver	4,340,467	2,436,245 (56.1)	11,232	1,898 (16.9)
Muscle	6,593,010	3,823,132 (58.0)	10,537	2,604 (24.7)
Total	14,096,080	7,910,143 (56.1)	26,414	5,813 (22.0)

* Reported only those eQTL regions that include at least 2 significantly associated variants.

† Percentage of the total number found in tissue.

Out of the 14,096,080 significant associations, 76.72% were classified as SNPs, 13.72% as insertions, and 9.56% as deletions. In total, 5,925,721 variants (23.4% of the 25 million) were associated with at least 1 gene in 1 tissue. Out of these, 29.5% were novel variants, as they were not described in the Ensembl database (Release 106: April 2022).

For each gene and within each tissue, an eQTL region was defined at ±1 Mb from any significantly associated polymorphism, merging them if they intersected. We considered for further analyses only the 26,414 eQTL regions that included at least 2 significant polymorphisms (Table 1). If we included the regions that were constituted by a single significant polymorphism, a total of 9,825, 28,429, and 23,409 eQTL regions would have been defined for duodenum, liver, and muscle, respectively. These 39.51–47.28% of eQTL regions constituted by a single significant polymorphism are indicative of putative spurious association signals, since linkage disequilibrium around a given location is expected to lead to a set of multiple significant signals.

### Distribution of *cis*- and *trans*-regulatory variants with respect to their eQTL and gene region

On average, 56.1% of the significantly associated variants were located at less than 1 Mb from their associated gene (i.e., *cis*-regulatory variants). However, this proportion was much lower regarding *cis*-eQTL regions (22.0%), indicating that *cis*-eQTL regions comprised more significant polymorphisms, but *trans*-eQTL regions were more abundant.

**Figure 2:**
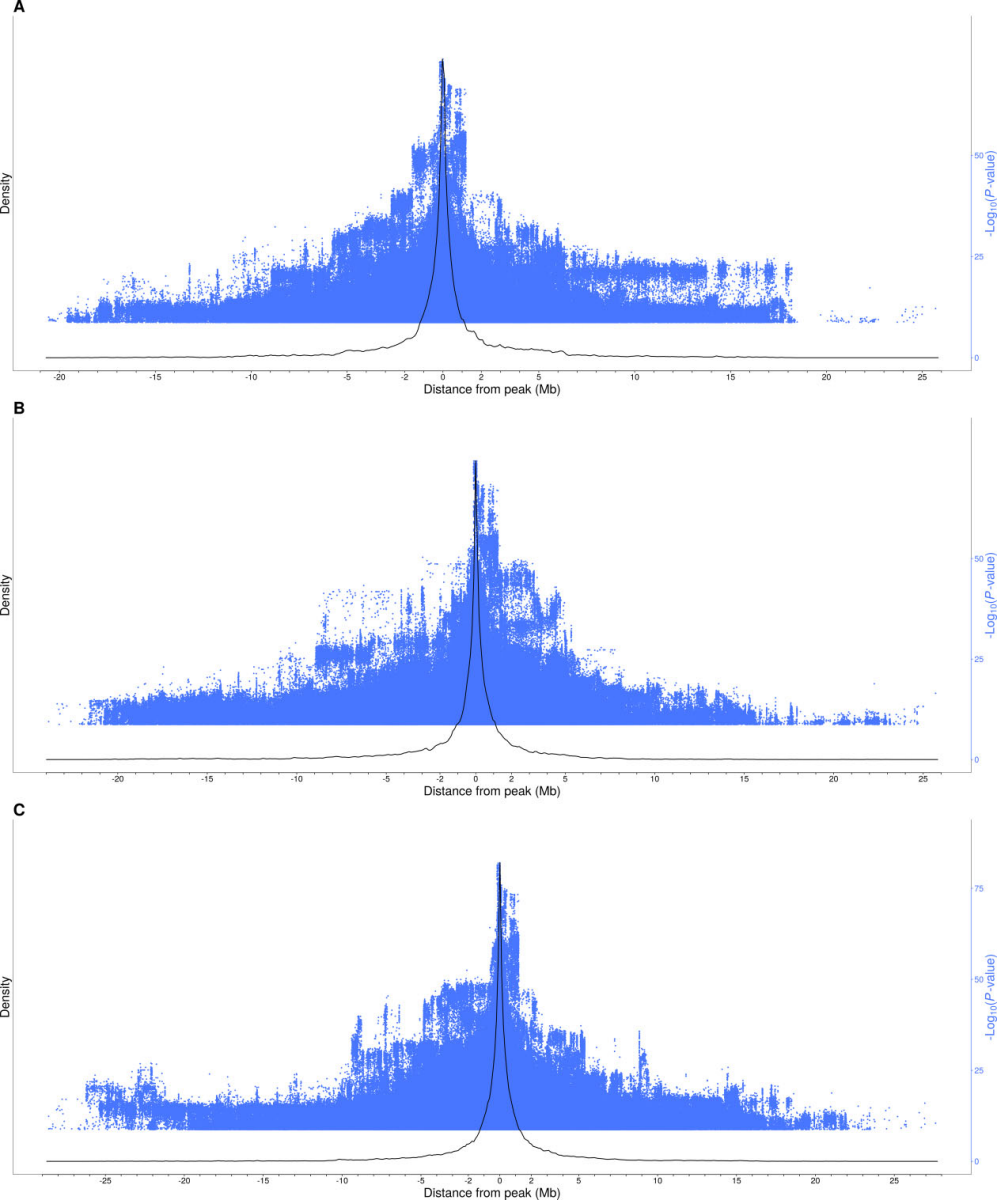
Density plot (black curve) representing the distance between each significantly associated polymorphism to the peak. The peak is defined as the most significant polymorphism of an eQTL region (or their mean, if multiple). The significance of the polymorphisms is provided in blue dots. Sexual and mitochondrial chromosomes have been excluded. (A) Duodenum. (B) Liver. (C) Muscle.

To evaluate the distribution of significant polymorphisms on each eQTL region, their distance to the top polymorphism (i.e., the smallest *P* value) was plotted for each tissue (Fig. 2). The density plot showed that most of the polymorphisms were at less than 1 Mb from the top polymorphism of their eQTL region. Therefore, the use of a window of ±1 Mb from the gene region to define a *cis*-regulatory variant seemed appropriate, as the linkage disequilibrium between a variant and the most significant polymorphism of its eQTL region rarely surpassed this 2-Mb window.

**Figure 3:**
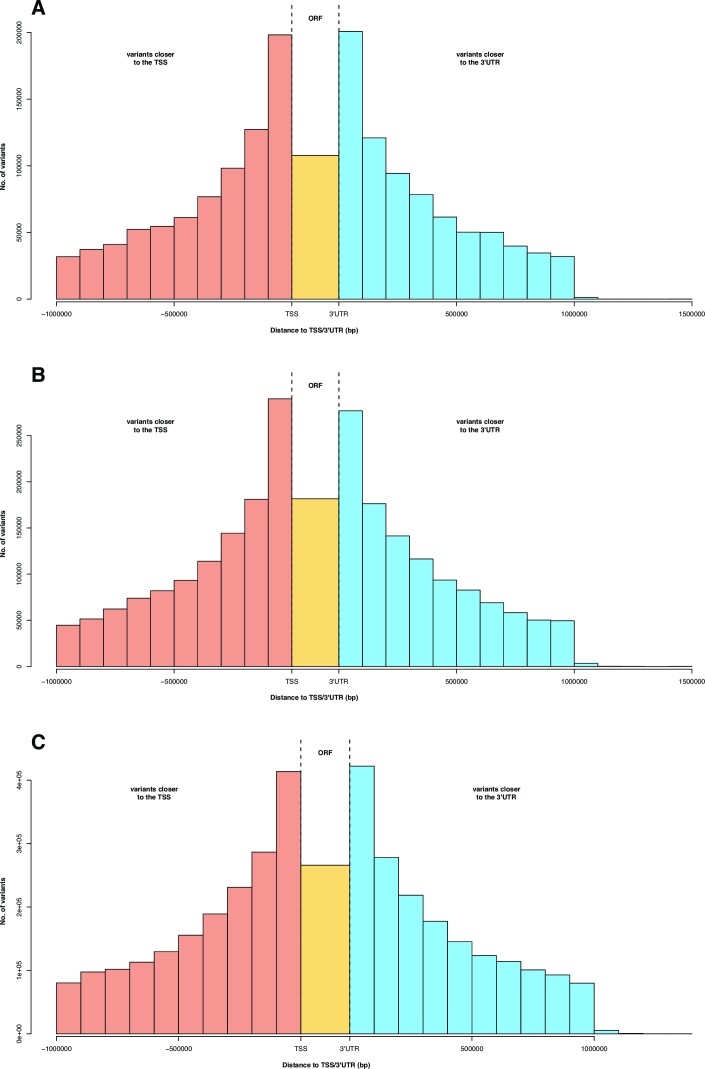
Distribution of *cis*-regulatory variants within their *cis*-eQTL region grouped by distance. The distance has been calculated between the position of each *cis*-regulatory variant and the position of the proximal transcription start site (TSS) or the 3′UTR (untranslated region) of their associated gene, whichever is closest. Those *cis*-regulatory variants that were located within the open reading frame (ORF) were placed between the TSS and the 3′UTR. (A) Duodenum. (B) Liver. (C) Muscle.

We then assessed the distribution of the defined *cis*-regulatory variants within a 1-Mb window prior to the start and posterior to the end sites of the studied genes (i.e., within the gene region +1 Mb on both sides). The same pattern of distribution was observed for the 3 tissues (Fig. 3). The regions with the greatest number of *cis*-regulatory variants were those located within 100 kb upstream the TSS and downstream the 3′UTR (untranslated region), whereas the number of *cis*-regulatory variants found within the open reading frame (ORF) of the gene was much lower.

**Figure 4:**
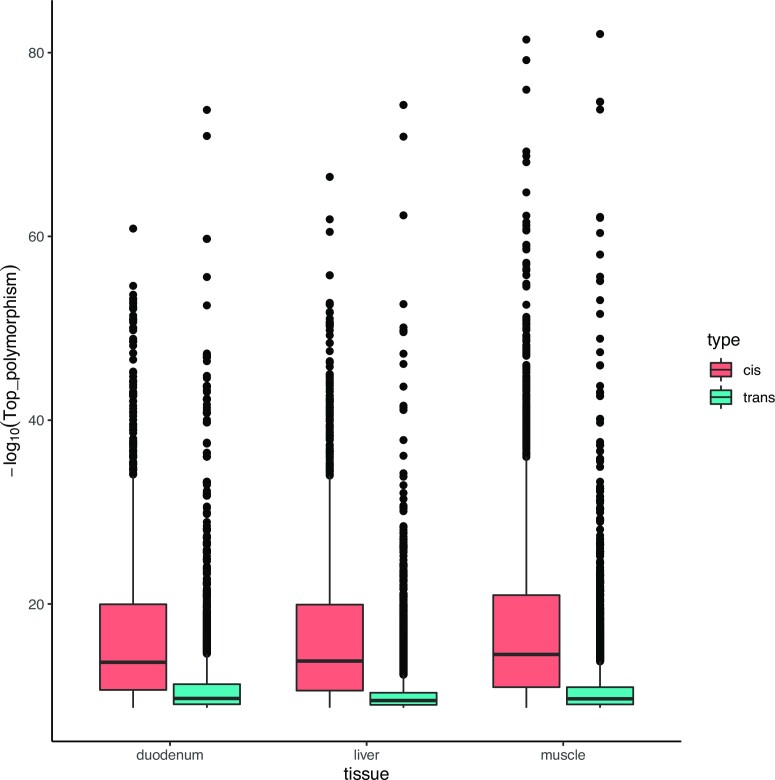
Comparison between the significance values of the top polymorphisms of *cis*-eQTL and *trans*-eQTL regions. Boxplots have been drawn for each tissue. Sexual and mitochondrial chromosomes have been excluded.

Unsurprisingly, the top polymorphisms found in *cis*-eQTL regions had lower *P* values than those found in *trans*-eQTL regions (Fig. 4). On average, 56.1% of the associations found were *cis*-regulatory variants, but only 22% of the annotated eQTLs were in *cis*. Therefore, most of the *cis*-eQTL regions were formed by a greater number of associated polymorphisms in linkage disequilibrium, whereas *trans*-eQTL regions, although numerous, had a lesser number of associated polymorphisms. Regarding the number of top polymorphisms found in common, 53 variants were associated with the same gene across the 3 tissues. Out of these, there were 6 *cis*-regulatory variants (Table 2).

**Table 2:** Top *cis*-regulatory variants found in common between duodenum, liver, and muscle eQTL regions

Associated gene		Top *cis*-regulatory variant			
EnsemblID	Gene name	Chromosome	Position	Reference allele	Alternative allele
ENSSSCG00000005103	*DET1*	1	190,113,347	T	TC
ENSSSCG00000013039	*NUDT22*	2	7,886,878	TC	T
ENSSSCG00000001398	*SLA-7*	7	24,180,079	C	CT
ENSSSCG00000011121	*CELF2*	10	60,516,892	C	A
ENSSSCG00000039915	*R3HCC1*	14	7,427,081	A	G
ENSSSCG00000028523	*HUS1*	18	48,525,415	T	C

### Hotspot and top-hotspot regulatory polymorphisms, predicted consequences on protein structure, and gene ontology analyses

A total of 5,183 hotspots (i.e., significant polymorphisms associated with the expression of 10 or more genes) were found in duodenum, 7,186 in liver, and 27,505 in muscle. Out of these, 110 hotspots in duodenum were predicted to have a moderate or high impact on the protein sequence, 133 in liver, and 452 in muscle (Supplementary Table S1). Besides, 102 of these hotspots were found in common across the 3 tissues and were located in 48 genes. Out of these, only 20 genes were simultaneously expressed on the 3 tissues, including 11 transcription factors and cofactors (AURKAIP1, HES4, NOC2L, TRIM28, ZNF134, ZNF274, ZNF544, and other 4 zinc finger proteins). Remarkably, these 102 hotspots shared among the 3 tissues were located within the same genomic region (56.3–64.5 Mb) on SSC6.

**Figure 5:**
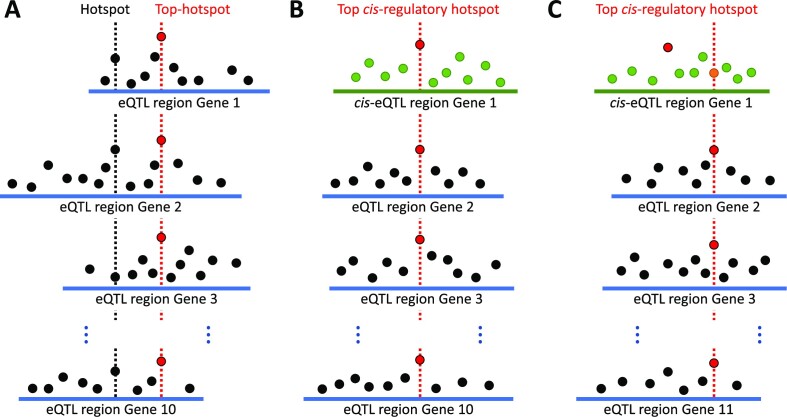
Definition of hotspot, top hotspot, and the 2 types of top *cis*-regulatory hotspots. Any polymorphism significantly associated with the expression of a particular gene is marked as a black dot, whereas *cis*-regulatory variants are marked in green. A red dot represents the most significantly associated polymorphism within an eQTL region. (A) A hotspot is defined as any polymorphism significantly associated with the expression of 10 genes or more; within them, those hotspots that are the most significantly associated polymorphism of at least 10 eQTL regions are defined as top hotspots. (B) Top *cis*-regulatory hotspots are top hotspots that are the most significantly associated polymorphism of their *cis*-eQTL region. (C) The other type of top *cis*-regulatory hotspots are top hotspots that are *cis*-regulatory variants (orange) but are not the most significant polymorphism of their *cis*-eQTL region.

Through the joint analysis of hotspots and eQTL regions, top hotspots can be defined as the most significantly associated polymorphism in at least 10 eQTL regions (Fig. 5A). Thus, 94,176 and 84 out of the total hotspots previously defined for duodenum, liver, and muscle were declared as top hotspots, respectively. In total, 23 top hotspots were the top c*is*-regulatory variant of 11 genes among the 3 tissues (Fig. 5B, Table 3, Supplementary Table S2). Due to being simultaneously a top hotspot and the most significant polymorphism of its *cis*-eQTL region, they had a strong likelihood of being the causal mutations with a potential role as regulatory variants. Note, however, that 22 additional top hotspots, across tissues, were *cis*-regulatory variants of another 11 genes without being the most significantly associated polymorphism of its *cis*-eQTL region (Fig. 5C, Supplementary Table S2). Altogether, 22 genes with 45 top *cis*-regulatory hotspots were postulated as regulators among the 3 tissues. Remarkably, some of these were already described as transcription factors and cofactors: ARL2BP, CHD7, CHD8, LHX6, and ZNF331 in liver and NFYC in muscle (Supplementary Table S2). By definition, and excluding those variants in complete linkage disequilibrium, some regulators had more than 1 top *cis*-regulatory hotspot, which were associated with the expression of a different number of genes.

**Table 3:** Top hotspots that were also the most significant polymorphisms of their *cis*-eQTL region

Tissue	EnsemblID	Gene name	Top *cis*-regulatory hotspot	No. of coexpressed genes
duodenum	ENSSSCG00000006561	*SLC39A1*	4:96,172,709_C/CT	39
liver	ENSSSCG00000005530	*LHX6*	1:262,892,119_T/C	20
liver	ENSSSCG00000023140	*EIF2B4*	3:112,449,895_C/CT*	27
liver	ENSSSCG00000006231	*CHD7*	4:72,782,568_C/CTTT	35
liver	ENSSSCG00000035772	*CDH5*	6:26,224,686_CT/C	15
liver	ENSSSCG00000031793	*ZNF331*	6:56,388,054_T/C*	21
liver	ENSSSCG00000003680	*RALBP1*	6:98,862,179_A/G; 6:98,862,186_A/G; 6:98,862,192_T/A; 6:98,862,193_T/G; 6:98,862,199_CT/C; 6:98,862,206_G/A; 6:98,862,214_C/T; 6:98,862,215_C/T	86
liver	ENSSSCG00000002127	*CHD8*	7:77,040,284_A/AA	92
liver	ENSSSCG00000031538	*RNASE4*	7:78,212,187_G/GTGTGTGTA	36
liver	ENSSSCG00000038622	*HS3ST3A1*	12:56,976,226_C/CCAAAAAAAAA	50
muscle	ENSSSCG00000039550	*EMC4*	7:79,160,941_GA/G; 7:79,246,475_A/G; 7:79,246,477_G/A; 7:79,561,343_T/C; 7:79,816,434_T/G; 7:79,816,437_G/GT	324

* Only significant polymorphism of its *cis*-eQTL region.

In order to evaluate the modulation of the expression of several genes through changes in the expression of a regulator gene, pathway, gene ontology, and coexpression analyses were carried out between the 22 genes postulated as regulators (i.e., with a top *cis*-regulatory hotspot) and the rest of the genes associated with the same regulatory variant in *trans*, henceforth referred as *trans*-associated genes.

Among the 22 regulator genes, 1 was a long noncoding RNA (lncRNA) and another 2 were novel genes with no known ortholog in humans. All the remaining 19 genes participated together with their *trans*-associated genes in at least 1 pathway. In general, most of the regulators were coexpressed with all their *trans*-associated genes. The data on biological functions and pathways, as well as coexpression results for the 22 genes with top *cis*-regulatory hotspots, are available in Supplementary Tables S3 and S4, respectively.

In duodenum, *SLC39A1* was the only putative regulator found, and it shared its role in transport and multicellular organism development pathways with 12 of its *trans*-associated genes. Remarkably, *SLC39A1* showed coexpression with all its 38 *trans*-associated genes, disregarding if they shared the same pathway.

The tissue with the greatest number of putative regulators was liver (14 genes). After the gene ontology analyses, the most relevant regulators based on the shared functions with their *trans*-associated genes were *CHD7*, *CHD8*, *CTSC*, and *RALBP1*. CHD7 and CHD8, which are chromodomain helicase DNA binding proteins, were involved in gene expression together with the other 19 and 27 *trans*-associated genes, respectively. Furthermore, they also participated in chromosome organization and animal organ and embryo development. In addition, *CHD7* was also involved in growth regulation together with the other 3 *trans*-associated genes. Despite the *cis*-polymorphism of *CHD8* being associated with 92 genes, *CHD8* was coexpressed with only 36% of them, while *CHD7* was coexpressed with all its 34 *trans*-associated genes. The *CTSC* gene was a regulator of multicellular organismal development along with the other 16 *trans*-associated genes, but it also participated in the regulation of the immune system process and in the response to organic substances. Intriguingly, *CTSC* was coexpressed with only 8 of its 49 *trans*-associated genes. Participating in a plethora of different functions, RALBP1 was a protein involved in the regulation of metabolic process together with the other 38 *trans*-associated genes, but it was also involved in phosphorylation, mitochondrion organization, and the regulation of GTPase activity and developmental processes. It is also worth noting that *RALBP1* showed coexpression with all of its 85 *trans*-associated genes.

Of the 4 regulators in muscle, *EMC4* was the gene with the greatest number of *trans*-associated genes. Out of its 323 *trans*-associated genes, *EMC4* was coexpressed with 314 (97%) of them. Among the pathways shared with 44 and 69 of them, *EMC4* participated in the apoptotic process and in organelle organization, respectively. *GCAT* was involved with the other 29 genes in the metabolism of amino acids and derivatives, and it was coexpressed with 37 of its 38 *trans*-associated genes. Although *NFYC* was only the potential regulator of other 10 *trans*-associated genes, it was coexpressed with 7 of them, and together with all but 1 of its *trans*-associated genes, was implicated in the cellular nitrogen compound metabolic process, similar to the aforementioned metabolism of amino acids. In addition, as a transcription factor, NFYC and 2 other *trans*-associated genes participated in the regulation of transcription by RNA polymerase II. In this context, *POLR2F*, the remaining regulator, encoded a subunit of RNA polymerase II and thus played a role in gene expression together with the 28 other *trans*-associated genes. *POLR2F* was also found in pathways related with cellular nitrogen compound biosynthetic process, developmental biology, and immunity but did not show a great percentage of coexpression with its *trans*-associated genes (16% and 67%, depending on the top *cis*-regulatory hotspot considered).

### Motifs in *cis*-regulatory SNPs

**Figure 6:**
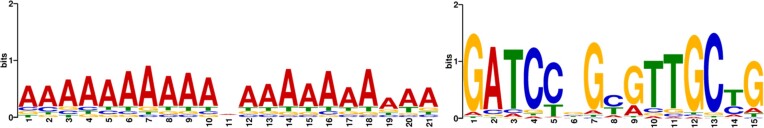
Sequence logo for the consensus DNA motifs found in common in the 3 tissues. Mutations were located in position 11 on the left motif and usually found in position 6 on the right motif.

Regions of 20 bp around the top SNPs in *cis*-eQTL regions (852 SNPs in duodenum, 1,142 SNPs in liver, and 1,605 SNPs in muscle) were selected to perform motif discovery using the MEME Suite. Two motifs were recurrently found in the 3 tissues (Fig. 6).

The first motif was composed of a poly(A) sequence or a poly(T) sequence if the reverse complement sequence was considered. This motif was found in 44 genes in duodenum, 79 in liver, and 103 in muscle (Supplementary Table S5).

The second motif was 5′-GATCCNGYGTTGCYG-3′, which was found in 22 genes in duodenum, 21 in liver, and 36 in muscle (Supplementary Table S5). Remarkably, a guanine or a cytosine was almost always found on the mutation site in the reference or the alternative sequence.

## Discussion

In the present study, we provide a catalog of eQTLs associated with the expression levels of local and distal transcripts in liver, duodenum, and muscle tissues. More than 9.68 × 10^11^ combinations between 25 million polymorphisms and 14,916 genes expressed in duodenum, liver, and muscle were tested following an eGWAS approach, resulting in 14,096,080 significant associations. After filtering out eQTL regions that contained a single associated polymorphism, the remaining significant associations were grouped into 26,414 eQTL regions.

Among these significant associations, only 443,857 polymorphisms (3.1%) were associated with the same gene across the 3 tissues, reflecting a high proportion of identified genetic variants with tissue-specific regulatory potential. This is in agreement with what has been observed in humans, as common regulatory variants are less abundant than tissue-specific variants, which are usually *trans*-regulatory variants [22]. However, although *cis*-regulatory variants are usually found in common across tissues with a lesser tissue specificity than *trans*-regulatory variants, we observed a similar ratio (3.2%) of shared *cis*-regulatory variants across the 3 tissues, which may be due to the limited relationship between the 3 tissues. Among the 3 analyzed tissues, muscle was the tissue with the greatest number of significant associations, followed by liver and duodenum. This difference in significant associations between muscle and the other 2 tissues may be due to the increased selective pressure that muscle has experienced over the past century, as lean meat percentage was one of the main traits considered in breeding programs [23]. Nowadays, pig breeds highly differ in muscle growth and structure [24, 25], which are in turn influenced by pre- and postnatal muscle cell expression [26, 27]. Although moderate overlapping of eQTLs between muscle and liver has already been reported in pigs [9], we observed the greatest number of shared significant associations between duodenum and liver (1,121,266). This may be due to the shared embryonic origin of the duodenum and liver tissues, which originate from the endodermal layer, whereas muscle has its origin in the mesodermal layer. Similar results were also reported in humans, where a strong correlation was observed between closely related tissues and shared eQTLs [22].

Since the locations of regulatory elements are not well defined for most genes, the definition of a window size for annotating eQTL regions varies across studies, from 10 kb in yeast to 20 Mb in mice [28]. Traditionally, most of the literature defines *cis*-eQTL regions as a 1-Mb window from the TSS of the target gene [4, 5, 7, 29, 30]. However, *cis*-regulatory variants can be located further than 1 Mb from the TSS [31]. In addition, *cis*-regulatory variants can also be found within the 3′UTR of genes [32], participating in the posttranscriptional regulation of gene expression by altering the binding sites of RNA molecules such as microRNAs or lncRNAs, among other mechanisms. Thus, in this study, we defined a *cis*-regulatory variant if it was located within the expanded gene region (i.e., the gene region ±1 Mb). In accordance with some studies that reported that most of the *cis*-regulatory variants were located within 100 kb from the TSS [15, 33], we observed the same pattern across duodenum, liver, and muscle. Nonetheless, the *cis*-regulatory variants located within 100 kb from the 3′UTR were as abundant as those located within 100 kb upstream the TSS, which implies that studies on regulatory variants should not be focused on the TSS neighboring region alone. As expected, the number of *cis*-regulatory variants located within ORFs was much lower than those located within 100 kb from the TSS and the 3′UTR.

In addition, as eQTL regions were annotated by intersecting the expression-associated variants that were located at less than 2 Mb, the most significant polymorphism of a *cis*-eQTL region could be located further than 1 Mb from its associated gene. The consideration of this 1-Mb window is supported by the observed linkage disequilibrium between an associated variant and the most significant polymorphism within an eQTL region, which rarely surpassed 2 Mb. Nonetheless, the definition of *cis*- and *trans*-eQTLs cannot be solely based on the distance to the associated gene, as this may change depending on the structure of the population used and the linkage disequilibrium between the variants under consideration. For example, the widest (60 Mb) eQTL region was located on SSCX, as the recombination rate in heterosomes is much lower than in autosomes.

Although the number of *cis*-eQTLs regions was lower than the number of *trans*-eQTL regions, *cis*-eQTL regions had a greater number of associated polymorphisms, and their most significant polymorphisms usually had lower *P* values. Previous studies in humans have already described weaker and more indirect effects of *trans*-regulatory elements on gene expression [4, 33]. Moreover, due to the multiple testing correction that must be applied, *trans*-eQTL regions with small effects are particularly difficult to detect [34]. However, defining hotspot regions can help investigate and determine the effect of *trans*-eQTL regions on gene regulation, as it avoids spurious associations and increases detection power.

Despite having the lowest number of annotated eQTL regions among the 3 tissues, the duodenum had the greatest number of annotated *cis*-eQTL regions. On the contrary, the liver had the greatest amount of annotated eQTL regions but the lowest percentage of *cis*-eQTL regions. This may be indicative of the complexity of the regulatory mechanisms in liver, which would be more dependent on *trans*-regulatory elements. Following this hypothesis, the amount of hotspot *cis*-regulatory variants was greatest in liver, whereas a single hotspot *cis*-regulatory variant was found in duodenum.

As previously mentioned, 3.2% of the *cis*-regulatory variants were shared between duodenum, liver, and muscle, and only 6 top *cis*-regulatory variants were found in common among the 3 tissues. In agreement with our findings, the existence of common regulatory elements across tissues has already been documented [5, 30, 35, 36], including shared *cis*-eQTLs between gluteus medius muscle and liver in a Duroc population [9]. Although the 6 *cis*-regulatory variants found in our study were the most significantly associated signal of their *cis*-eQTL regions, indicating a great potential to be the causal mutations and explain the variability in expression of their associated genes, other polymorphisms in linkage disequilibrium with them should not be discarded.

Through the modification of expression levels of their associated genes, the 6 top *cis*-regulatory variants found across the 3 tissues have the potential to determine production and health traits in pigs. *CELF2* was suggested as a candidate gene for backfat thickness in Large White pigs [37]. *HUS1* and *NUDT22* were related to the intramuscular fat content in pigs [38, 39]. In addition, *HUS1* was also proposed as a candidate gene for meat color in muscle from Duroc × Luchuan pigs [7] and for the abundance of alanine aminotransferase in blood from Large White pigs [40]. After an *in vitro* infection with *Salmonella*, *R3HCC1* was downregulated in porcine neutrophils [41]. In blood from the same Large White pig population as that used in this study, a *cis*-regulatory variant was also associated with the expression of *SLA7* [8]. The *cis*-regulatory variant (rs80859275) was located at ∼478 kb from the most significant signal found in our data across the 3 tissues. Thus, it could be in linkage disequilibrium with the potential causal mutation reported by our study, extending its regulatory role to 4 tissues (blood, duodenum, liver, and muscle). The importance of *SLA-7* lies in its participation on the porcine major histocompatibility complex, swine leukocyte antigen, although its exact functions remain to be determined [42].

Among the putative causal mutations on hotspot *trans*-regulators (i.e., polymorphisms on the coding region of a gene that were significantly associated with the expression of more than 10 genes in *trans*), 3 missense mutations associated with the expression of 33 genes in *trans* were found on the coding region of the *NOC2L* gene. NOC2L is a transcription factor that inhibits the histone acetyltransferase activity and prevents all core histones from being acetylated [43]. On the coding region of another gene, *TRIM28* had 2 predicted missense mutations that were associated with a total of 29 genes in *trans*. This transcriptional regulator acts as a repressor of gene expression by recruiting CHD3 [44]. TRIM28 also participates in host innate immune response [45] and negatively regulates aggresome formation [46], among several other functions. In pigs, *TRIM28* knockdown in gestating sows produced epigenetic variations in their embryos, including those of the promoter region of the *IGF2* gene, affecting their developmental processes [47].

Based on the genes with top *cis*-regulatory hotspots, 22 were postulated as regulators. Out of this list, it is worth mentioning that *SLC39A1*, the only regulator found on duodenum and coexpressed with all of its *trans*-associated-genes, was not previously described as a transcription factor or cofactor in the literature but rather as a regulator of zinc homeostasis in the gut [48]. Both CHD7 and CHD8 are members of the chromodomain-helicase-DNA binding protein family and participate in transcription regulation by chromatin remodeling [49]. However, their interpretation as liver-specific transcriptional regulators in our study is obscured, as both *CHD7* and *CHD8* are ubiquitously expressed, but no top *cis*-regulatory hotspots were described for them in the other 2 tissues of our study. Nonetheless, human patients with a lower expression of *CHD8* have a favorable prognosis in liver cancer [50]. Apart from participating in immunity, the expression of *CTSC* was also associated with feed efficiency and loin tenderness in pigs [51, 52]. In muscle, NFYC was the only regulator previously described as a transcription factor. NFYC binds to 5′-CCAAT-3′ motifs and participates in muscle cell differentiation [53]. In pigs, *NFYC* was reported as coassociated with lean percentage and average daily gain in a regulatory gene network [54]. The gene with the greatest number of coexpressed genes was *EMC4*. Despite *EMC4* being expressed in the 3 tissues, its hotspot regulatory role was observed only in muscle. The protein encoded by this gene is mainly located in the endoplasmic reticulum membrane and participates in the development of muscle [55, 56].

We found across the 3 tissues the same 2 motifs using the sequences surrounding the most significant SNPs in *cis*-eQTLs. The first of them, a poly(A) motif, was a short tandem repeat (STR) of adenines that could also include few other bases. Adenine-rich STRs are the most common sequences found in the human genome [57], and their potential role as transcriptional regulators may be due to a variety of reasons. For example, some of the sequences were similar to the 5′-AAUAAA-3′ endonucleolytic cleavage site prior to the addition of the poly(A) tail [58]. However, not all of the significantly associated polymorphisms were located in the 3′ flanking region. Hence, other mechanisms in which poly(A) or poly(T) could affect the expression of large RNA molecules were the T-loop RNA folding motif [59] and nucleosome control and accessibility [60, 61]. The second motif, 5′-GATCCNGYGTTGCYG-3′, was usually found in the 5′ flanking region of the associated genes. Its potential regulatory role was also supported by the fact that the mutated base in the reference or the alternative sequence was almost always a guanine or a cytosine and thus possibly impairing a CpG site. In agreement with its potential role as a regulator, the same motif was found in the promoter regions of human genes, despite the fact that no transcription factor was found to bind on such sequences.

## Conclusions

In conclusion, we have reported more than 14 million significant associations between 5,925,721 variants and the expression of 10,019 genes in duodenum, muscle, and liver, which are publicly available. We have also reported that most of the *cis*-regulatory variants were equally abundant within the 100 kb upstream the TSS or 100 kb downstream the 3′UTR. In addition, our results have allowed the identification of genomic *cis*, *trans*, and hotspot regions associated with the expression of such genes within and across tissue, which will shed light on the molecular mechanisms of regulatory variations that shape end-trait phenotypes.

## Material and Methods

### Ethics statement

Since the pigs analyzed in this study were not subjected to any experimental procedures given that samples were taken postmortem, the study was exempt from the European Union Directive 2010/63/EU about the protection of animals used in experimentation. Duroc and Landrace pigs were reared and slaughtered in a commercial farm and abattoir following Spanish national and institutional guidelines for Good Experimental Practices. Large White pigs were reared and slaughtered according to procedures approved by the French Veterinary Services at INRAE UE3P France Génétique Porc phenotyping facilities (user establishment agreement number C-35–240–7; UE3P [62]).

### Animal material

A total of 300 pigs of 3 different breeds (*n* = 100 Duroc, *n* = 100 Landrace, and *n* = 100 Large White) were used in this study. Duroc animals were distributed in 3 batches balancing gender, 50 males and 50 females, and belonged to 33 litters obtained from 33 sows and 10 boars. Large White pigs were distributed in 4 batches of uncastrated males and belonged to 84 litters obtained from 84 sows and 43 boars. Landrace animals were taken from 1 batch, 39 males and 61 females, and belonged to 74 litters obtained from 74 sows and 18 boars. Each breed was raised in a different farm and fed *ad libitum* with a commercial cereal-based diet. Pigs were slaughtered at 5 to 7 months of age in a commercial abattoir, and blood, duodenum, liver, and muscle samples were collected. Full information on the animal material is publicly available on the FAANG data portal (accession number requested).

### DNA and RNA extraction and sequencing

Genomic DNA was extracted using the NucleoSpin Blood kit (Macherey-Nagel, Düren, Germany) on blood samples from Duroc and Landrace pigs and on liver samples from Large White pigs using the QIAamp DNA Mini Kit (Qiagen, Hilden, Germany). Duodenum, liver, and muscle samples were homogenized using biodisruptor and bead tubes (Lysing matrix D). Duodenum and liver RNA was extracted using a chemagic 360 instrument with RNA Tissue10 Kit H96 (PerkinElmer, Baesweiler, Germany). Muscle RNA was extracted using the RiboPure RNA Purification Kit (Invitrogen, Carlsbad, CA, USA) and RNeasy Fibrous Tissue Mini Kit (Qiagen). Detailed protocols are publicly available on the FAANG data portal [63, 64]. DNA and RNA were quantified in a NanoDrop ND-1000 spectrophotometer (NanoDrop Technologies, Wilmington, DE, USA). Purity and integrity of RNA was assessed in a Bioanalyzer-2100 (Agilent Technologies, Santa Clara, CA, USA). RNA integrity number (RIN) values ranged from 6 to 9.2 for duodenum and liver samples, while muscle samples had RIN >8 values. For sequencing, more than 2 µg total RNA in a concentration range of 50 to 200 ng/µL was provided. Libraries were prepared using the TruSeq Stranded mRNA Sample Preparation kit (Illumina, San Diego, CA, USA). WGS libraries were constructed with an insert size of 470 bp, whereas the insert size of transcriptomic libraries was 285 bp. All DNA samples (*n* = 300) were sequenced with a yield >30 Gb, resulting in a WGS depth of about 10×. For RNA sequencing, duodenum, liver, and muscle samples (*n* = 900) were sequenced with a depth of >90 million reads. All samples were paired-end sequenced (2 × 150 bp) in an Illumina NovaSeq6000 platform (RRID:SCR_020150) at Centro Nacional de Análisis Genómico (CNAG-CRG, Barcelona, Spain).

### Mapping and annotation of DNA and RNA reads

Quality of raw DNA and RNA sequenced reads was assessed with the FastQC (RRID:SCR_014583) software [65]. DNA sequences were mapped against the reference genome (*Sscrofa11.1* assembly) with BWA-MEM/0.7.17 [66]. Alignment files containing only properly paired, uniquely mapping reads without duplicates were processed using Picard (RRID:SCR_006525) to add read groups and to remove duplicates.

Genetic variant calling was conducted with GATK (RRID:SCR_001876)/4.1.8.0 HaplotypeCaller [67] to extract SNPs and indels from whole-genome sequences. HaplotypeCaller was used with the default parameters with the exception of setting the “–minimum-mapping-quality” to 20. Then, joint genotyping was carried out with GATK/4.1.8.0 CombineGVCFs to obtain a multisample gVCF file. Thereafter, BCFtools (RRID:SCR_005227)/1.9 norm [68] was used to split multiallelic sites (SNPs and indels) into multiple rows. For downstream analyses, genetic variants were filtered if the minor allele frequency was below 5% and/or if there was more than 10% missing genotype data using PLINK (RRID:SCR_001757)/v1.90b3.42 [69].

RNA sequences were mapped against the reference genome (*Sscrofa11.1* assembly) and the Ensembl Genes 101 annotation database with STAR (RRID:SCR_004463)/v2.5.3a [70], and counts were quantified with RSEM (RRID:SCR_013027)/1.3.0 [71]. During the filtering step, lowly expressed genes (counts per million [cpm] <10/minimum library size in millions) and those missing in more than 20% of the animals were removed. Then, within-tissue counts were normalized by trimmed mean of M values and transformed to cpm using log_2_ and a prior count of 1 with the cpm function of the edgeR (RRID:SCR_012802)/3.30.3 Bioconductor package [72]. In addition, to avoid normalization artifacts, raw counts with a value of 0 were replaced by NA.

The distribution of each normalized dataset was assessed by applying the Shapiro–Wilk test to each expressed gene following a leave-one-out procedure (i.e., taking out a sample and conducting the normality tests in the remaining 299 samples). Two samples from duodenum and 3 from muscle significantly reduced the number of genes with approximately normally distributed expressions values and were thus considered outliers and removed from the analyses.

### eGWAS

For each of the 3 tissues, eGWAS were carried out between the filtered polymorphisms and the normalized expression data by applying the following model with the fastGWA tool from GCTA/1.93.2 [73]:


\begin{eqnarray*}
{y}_{hijk} = se{x}_{hj} + bree{d}_{hk} + {u}_{hi} + {s}_{il} \cdot {a}_{hl} + {e}_{hijk}
\end{eqnarray*}

where *y_hijk_* corresponds to the expression of the *h*th gene in the *i*th individual of sex *j* and belonging to the *k*th breed; *sex_hj_* corresponds to the *j*th sex effect (2 levels); *breed_hk_* corresponds to the *k*th breed effect (3 levels); *u_hi_* is the infinitesimal genetic effect of the individual *i*, with *u*∼MVN(0,G∙*σ*^2^*_u_*), where G is the genomic relationship matrix calculated using the filtered autosomal polymorphisms as described in [74] and *σ*^2^*_u_* is the additive genetic variance to be estimated; *s_il_* is the genotype (coded as 0, 1, or 2) for the *l*th polymorphism; *a_hl_* is the allele substitution effect of the *l*th polymorphism on the expression level of the *h*th gene; and finally, *e_hijk_* is a residual error term. Then, Bonferroni correction was applied to calculate genome-wide significance thresholds using the p.adjust function from the stats/4.0.4 R base package. Only those associations with an adjusted *P* value ≤0.05 were considered significant.

### eQTL clustering and consequence prediction

Preliminary eQTL regions were considered by clustering the significant polymorphisms at a distance of less than 2 Mb from each other. To reduce the number of false positives, only eQTL regions with a minimum of 2 polymorphisms were retained. Then, eQTL regions were extended 1 Mb on each side of the previously defined regions. Gene positions were extracted with the BioMart tool [75] from the Ensembl Genes 101 annotation database. Significant polymorphisms that were located at less than 1 Mb from their associated gene were defined as *cis*-regulatory variants. Therefore, eQTL regions containing a *cis*-regulatory variant were considered *cis*-eQTL regions. The remaining regions were considered *trans*-eQTL regions.

Functional predictions of the significant polymorphisms were performed with the Variant Effect Predictor tool [76] on the Ensembl Genes 106 annotation database.

### Hotspot and top-hotspot polymorphism definitions and network analysis

In the context of this study, a hotspot was defined as a polymorphism associated with the expression of at least 10 genes. Further, a top hotspot was defined as any hotspot that was the most significantly associated polymorphism (smallest *P* value) in at least 10 eQTL regions. Genes with associated top hotspots were checked for transcription factors and cofactors in the AnimalTFDB (RRID:SCR_001624)/v3.0 [77]. Top-hotspot regulatory polymorphisms that were classified as *cis*-regulatory variants were further studied through network analysis. For that purpose, we extracted all genes that were significantly associated with the same top hotspot, including the regulator gene associated in *cis*, and identified overrepresented gene ontology terms and KEGG pathways with the ClueGO plugin [78] from Cytoscape (RRID:SCR_003032) [79]. In addition, coexpression between each regulator and its *trans*-associated genes was assessed with the partial correlation and information theory approach [80], a network-based approach that combines partial correlation coefficient with information theory to identify significant correlations between each possible combination of genes.

### Motif discovery

As motif discovery in variable sequences is made difficult by the presence of elements such as indels, only SNPs that were top *cis*-regulatory variants were considered for the analysis. Two sequences ±10 bp of the position of the *cis*-SNP were extracted from the reference genome (*Sscrofa11.1* assembly), one including the reference SNP and the other with the alternative SNP. For each tissue, the 2 sets of sequences with the reference and the alternative alleles were submitted together to the MEME Suite (RRID:SCR_001783)/5.4.1 web tool [81] to perform motif discovery through the MEME tool [82]. Default parameters were used, but the maximum number of motifs to be searched was set to 15. Then, relevant consensus motifs were scanned against the same dataset with the FIMO tool [83] to assess the number of occurrences in a given tissue. Only those hits with a *q* value ≤0.1 were considered significant.

## Data Availability

The raw sequence data that support the findings of this study have been deposited in the FAANG data portal with the BioProject accession codes PRJEB58030 and PRJEB58031. The results of the eGWAS across tissues have been made publicly available on the following repository [84]. All supporting data and materials are available in the *GigaScience* GigaDB database [85].

## Additional Files

**Supplementary Table S1**. List of hotspot regulatory polymorphisms that could have a moderate or high impact on the protein structure and their associated genes.

**Supplementary Table S2**. List of hotspot regulatory polymorphisms that were *cis*-regulatory variants and their associated genes.

**Supplementary Table S3**. ClueGO results for each regulator gene with a top hotspot in *cis* that was associated with the expression of more than 10 genes in *trans*. Only the pathways where the regulator gene is present are included.

**Supplementary Table S4**. Coexpression results for the 22 genes with hotspot *cis*-regulatory variants.

**Supplementary Table S5**. FIMO results for the 2 motifs found and the distance between the mutation and the proximal TSS and 3′UTR of their associated gene.

## Abbreviations

3′UTR: 3′ untranslated region; bp: base pair; cpm: counts per million; eGWAS: expression genome-wide association studies; eQTLs: expression quantitative trait loci; GWAS: genome-wide association studies; KEGG: Kyoto Encyclopedia of Genes and Genomes; lncRNA: long noncoding RNA; ORF: open reading frame; STR: short tandem repeat; TSS: transcription start site; WGS: whole-genome sequencing.

## Funding

This project is part of GENE-SWitCH (https://www.gene-switch.eu) and has received funding from the European Union's Horizon 2020 Research and Innovation Programme under the grant agreement n° 817998. It is also part of EuroFAANG (https://eurofaang.eu), a synergy of 5 Horizon 2020 projects that share the common goal to discover links between genotypes and phenotypes in farmed animals and meet global FAANG objectives. Y.R.-C. was financially supported by a Ramon y Cajal contract (RYC2019-027244-I) from the Spanish Ministry of Science, Innovation and Universities. Some of the authors belonged to a Consolidated Research Group AGAUR, ref. 2021-SGR-01552.

## Competing Interests

The authors declare that they have no competing interests.

## Authors’ Contributions

M.B. designed the study. M.B., M.-J.M., M.C.A.M.B., and A.E.H. supervised the generation of the animal material used in this work. H.A., O.G.-R., M.M., M.-J.M., Y.R.-C., and M.B. performed the sampling. H.A., O.G.-R., M.M., and M.B. performed the DNA and RNA extractions. D.C.-P. performed the bioinformatic analyses. Y.R.-C. performed the coexpression analysis. D.C.-P., Y.R.-C., J.P.S., and M.B. analyzed the data and interpreted the results. D.C.-P. and M.B. wrote the manuscript. All authors read and approved the submitted version of the manuscript.

## Supplementary Material

giad042_Supplemental_Files

giad042_GIGA-D-22-00301_Original_Submission

giad042_GIGA-D-22-00301_Revision_1

giad042_Response_to_Reviewer_Comments_Original_Submission

giad042_Reviewer_1_Report_Original_Submission Samuele Bovo, Ph.D -- 1/24/2023

giad042_Reviewer_2_Report_Original_Submission Zhong-Yin Zhou -- 2/3/2023
