# Supplementary material for: Identification of transcriptional regulatory variants in pig duodenum, liver, and muscle tissues
Source: Gigascience. 2023 Jun 24;12:giad042. doi: 10.1093/gigascience/giad042 (PMC10290502; doi:10.1093/gigascience/giad042)
Supplement: giad042_GIGA-D-22-00301_Revision_1 [file giad042_giga-d-22-00301_revision_1.pdf]

GigaScience

Identification of transcriptional regulatory variants in pig duodenum, liver and muscle tissues

--Manuscript Draft--

**Manuscript Number:**	GIGA-D-22-00301R1
**Full Title:**	Identification of transcriptional regulatory variants in pig duodenum, liver and muscle tissues
**Article Type:**	Research
**Funding Information:**	Horizon 2020 (817998) — Not applicable<br>Ministerio de Ciencia, Innovación y Universidades (RYC2019-027244-I) — Dr. Yuliaxis Ramayo-Caldas<br>Agència de Gestió d'Ajuts Universitaris i de Recerca (2021-SGR-01552) — Not applicable
**Abstract:**	Background<br>In humans and livestock species, genome-wide association studies (GWAS) have been applied to study the association between variants distributed across the genome and a phenotype of interest. To discover genetic polymorphisms affecting the duodenum, liver, and muscle transcriptomes of 300 pigs from three different breeds (Duroc, Landrace and Large White), we performed expression GWAS between 25,315,878 polymorphisms and the expression of 13,891 genes in duodenum, 12,748 genes in liver, and 11,617 genes in muscle.<br>Results<br>More than 9.68×1011 association tests were performed, yielding 14,096,080 significantly associated variants, which were grouped in 26,414 expression quantitative trait locus (eQTL) regions. Over 56% of the variants were within 1Mb of their associated gene. In addition to the 100kb region upstream of the transcription start site, we identified the importance of the 100kb region downstream of the 3'UTR for gene regulation, as most of the cis-regulatory variants were located within these two regions. We also observed 39,874 hotspot regulatory polymorphisms associated with the expression of 10 or more genes that could modify the protein structure or the expression of a regulator gene. In addition, two motifs (5'-GATCCNGYGTTGCYG-3' and a poly(A) sequence) were enriched across the three tissues within the neighbouring sequences of the most significant SNPs in each cis-eQTL region.<br>Conclusions<br>The 14 million significant associations obtained in this study are publicly available and have enabled the identification of expression-associated cis-, trans- and hotspot regulatory variants within and across tissues, thus shedding light on the molecular mechanisms of regulatory variations that shape end-trait phenotypes.
**Corresponding Author:**	Daniel Crespo-Piazuelo<br>Institut de Recerca i Tecnologia Agroalimentàries<br>Caldes de Montbui, Catalunya SPAIN
**Corresponding Author Secondary Information:**	
**Corresponding Author's Institution:**	Institut de Recerca i Tecnologia Agroalimentàries
**Corresponding Author's Secondary Institution:**	
**First Author:**	Daniel Crespo-Piazuelo
**First Author Secondary Information:**	
**Order of Authors:**	Daniel Crespo-Piazuelo<br>Hervé Acloque<br>Olga González-Rodríguez

Powered by Editorial Manager® and ProduXion Manager® from Aries Systems Corporation

	Mayrone Mongellaz
	Marie-José Mercat
	Marco C.A.M. Bink
	Abe E. Huisman
	Yuliaxis Ramayo-Caldas
	Juan Pablo Sánchez
	Maria Ballester
**Order of Authors Secondary Information:**	

Response to Reviewers:

We want to thank the reviewers for their insight and suggestions. Their feedback has been really helpful in improving the manuscript. Below, each commentary has been addressed separately.

Reviewer reports:
Reviewer #1: The manuscript of Crespo-Piazuelo et al. describes eQTL discovery in pigs by merging WGS and RNA-seq data. The authors carried out WGS in about 300 pigs from three populations (LW, DU and LA) and the characterization of the transcriptome from three different tissues (duodenum, liver and muscle). After the detection of associated variants, followed their evaluation as cis- or trans-regulatory elements as well as hotspots elements; genes associated with the same top-hotspot underwent to over-representation analyses (GO terms and KEGG pathways) and co-expression analyses of trans-associated-genes. Overall, the manuscript presents a proper introduction, interesting results and a proper discussion. Protocols, data and results have been properly released through the FAANG data portal. The manuscript can be accepted in the present from. I have only some minors related to the material and methods part:

- Line 114-115. To evaluate the goodness of the obtained dataset, it would be nice to report the Ts/Tv value.
The Ts/Tv value of the dataset was 2.01, which is good overall. The value has been included in the revised version of the manuscript (L117).

- Line 466. Please, provide info about the sex of animals and their relatedness.
Duroc animals were distributed in three batches balancing gender, 50 males and 50 females, and belonged to 33 litters obtained from 33 sows and 10 boars. Large-White pigs were distributed in 4 batches of uncastrated males and belonged to 84 litters obtained from 84 sows and 43 boars. Landrace animals were taken from one batch, 39 males and 61 females, and belonged to 74 litters obtained from 74 sows and 18 boars. This information has been added in the final version of the manuscript (L467-472).

- Line 492-499. It is not clear how many Gbp have been produced for each WGS and RNA-seq data. "Whole genome (>30Gb; n=300)" means that each genome was covered by at least 30Gbp resulting in a depth of sequencing of about 10X?
All DNA samples were sequenced with a yield > 30 Gb, resulting in a whole genome sequencing (WGS) depth of about 10X, the mean yield for all samples was 45.122 Gb with a standard deviation of 9.72.
For RNA-seq, all samples were sequenced with a minimum depth of 45 M PE reads (2x150 bp) per sample (>90M reads). The mean yield for muscle RNA samples was 23.851 Gb with a standard deviation of 7.48; for liver samples was 22.631 Gb with a standard deviation of 5.82 and 22.00 Gb with a standard deviation of 5.73 for duodenum samples.
We have modified the sentence to clarify the sequencing depth for each WGS and RNA-seq data (L499-501).

- Line 494. Insert size of the library is missing.
Whole genome sequencing and transcriptomic libraries were constructed with 470 bp and 285 bp insert sizes, respectively. These values have been included in the final version of the manuscript (L497-499).

- Line 503-507. Did you use HaplotypeCaller default parameters?

The only exception that we applied to the default parameters was increasing the minimum-mapping-quality from 10 to 20. This has been clarified on the manuscript (L512-513).

After calling SNPs/INDELs, GATK best practices suggest some filtering recommendations. Did you make use of any variant recalibration or hard-filtering?
First, bcftools norm was used to split multiallelic sites (SNPs and indels) into multiple rows. Then, variants were flagged following GATK best practices for hard-filtering with the VariantFiltration tool. However, despite all the hard-filtering applied, at the end, only 0.8% out of the 25 million variants that passed the --maf 0.05 and --geno 0.1 PLINK filters were flagged variants. Thus, we considered that these flagged variants could be relevant as they were segregating in our population, and as such, we did not include GATK hard-filtering in the manuscript.

Did you retain only bi-allelic variants? Is the 5% used to retain a variant within breed or at the population level (in the second case, you are losing some variants).
We did not exclusively retain bi-allelic variants. We kept tri-allelic polymorphisms or variants such as microsatellites, albeit per notation and for the eGWAS, they were included and analysed as different variants against the reference on the same position. About 10.5% of the 25M polymorphisms shared the same position.
The reviewer is correct on the assertion that some variants were lost because of the 5% applied at population level for retaining a variant. However, we indeed tried the 5% filter at breed level on our first iteration, which is translated as 1.67% at population level, but this caused the eGWAS to be more prone to population structure artifacts. Therefore, as we were more focused on the common elements between breeds, we decided on applying the filtering at population level.

- Line 527. The GWAS model considers, for each tissue, the breed as covariate. What is the choice of this approach instead of running breed-specific GWASs followed by a meta-GWAS? Moreover, it would be nice to have, as results for the associated variants, the allele frequencies stratified by breed (not only at the level of the whole population).
The decision on analysing the samples in this fashion followed two main points. Speed was one of the main factors that moved us to analyse such data in such way. Performing each eGWAS per breed would have required thrice as much power computing and storage that with a merged dataset. In addition, it would have compromised the comprehension of the manuscript, as it would have required the discussion of each set in particular. Following this idea is the other main point, as we wanted to exclusively focus on those elements that were present in all the three breeds, that is why we chose to remove those genes that were missing in more than 20% of the animals. Nonetheless, we are considering the publication of a follow-up article that will analyse each breed individually using multi-tissue networks.

Reviewer #2: Crespo-Piazuelo et al collected duodenum, liver, and muscle tissues of 300 pigs from three different breeds (Duroc, Landrace and Large White), and performed whole genome and transcriptome sequencing to obtain eQTL regions. This is a huge project in pig breeding field. The study is very interesting especially the analysis of hotspot regulatory sits. However, I have several concerns for this manuscript.

1. The authors should integrate the GWAS or QTL data of economic phenotypes of pigs to improve the level of this manuscript.
We concur with the reviewer’s opinion that the inclusion of economically important traits to the manuscript would be of importance for the field. However, this study, as part of the GENE-SWitCH European project, was more focused on studying the transcriptional regulatory elements of the genomes, rather than determining their relationship to few specific complex traits. In addition, the few overlapped recorded traits for the 300 animals are being used on other manuscript of our partners of the GENE-SWitCH European project to evaluate genomic predictions with the inclusion of transcriptomic data. Altogether, we are limited on the inclusion of such measurements on this manuscript.

2. Taking "Identification of transcriptional regulatory variants in pig duodenum, liver and

	muscle tissues" as paper title might be better.<br>Thank you for the suggestion, we agree with the reviewer and have amended the title as recommended.<br><br>3. Promoters, enhancers et al. are "cis-regulatory elements". It's better to change cis-regulatory elements to cis-regulatory SNPs or variations.<br>It is true that the word "cis-regulatory elements" may refer to any sort of loci or regions with a regulatory role over the gene. Hence, to avoid any misleading, we have used the term "cis-regulatory variants" instead as suggested.<br><br>4. The analysis of regulatory elements should incorporate the data sets of ChIP-seq and/or STARR-seq.<br>These two datasets have been generated by different partners of the GENE-SWitCH European project. Likewise, the manuscripts with this information will be made publicly available in other publication(s). In addition, the animals used to generate both datasets (ChIP-seq and STARR-seq) were different from those used in this study, differing in age and breed (only Durocs were used). Thus, we are unable to incorporate this information to the manuscript.<br><br>5. The English writing of the manuscript is of a little poor quality. The manuscript should be carefully proofread to improve the quality of writing.<br>Following the reviewer suggestion, the manuscript was proofread by a native English speaker, our colleague Dr. Andrea Rau. This has been reflected in the acknowledgements section.
**Additional Information:**	
**Question**	**Response**
Are you submitting this manuscript to a special series or article collection?	No
**Experimental design and statistics**<br><br>Full details of the experimental design and statistical methods used should be given in the Methods section, as detailed in our Minimum Standards Reporting Checklist. Information essential to interpreting the data presented should be made available in the figure legends.<br><br>Have you included all the information requested in your manuscript?	Yes
**Resources**<br><br>A description of all resources used, including antibodies, cell lines, animals and software tools, with enough information to allow them to be uniquely identified, should be included in the Methods section. Authors are strongly encouraged to cite Research Resource Identifiers (RRIDs) for antibodies, model organisms and tools, where possible.	Yes

Have you included the information requested as detailed in our Minimum Standards Reporting Checklist?	
**Availability of data and materials**<br><br>All datasets and code on which the conclusions of the paper rely must be either included in your submission or deposited in publicly available repositories (where available and ethically appropriate), referencing such data using a unique identifier in the references and in the "Availability of Data and Materials" section of your manuscript.<br><br>Have you have met the above requirement as detailed in our Minimum Standards Reporting Checklist?	Yes

Powered by Editorial Manager® and ProduXion Manager® from Aries Systems Corporation

Manuscript

Click here to access/download;Manuscript;Crespo-Piazuelo_revised-May 22.docx

Identification of transcriptional regulatory variants in pig duodenum, liver and muscle tissues

Daniel Crespo-Piazuelo[1,*], Hervé Acloque[2], Olga González-Rodríguez[1], Mayrone Mongellaz[2], Marie-José Mercat[3], Marco C.A.M. Bink[4], Abe E. Huisman[5], Yuliaxis Ramayo-Caldas[1], Juan Pablo Sánchez[1], Maria Ballester[1,*]

[1]Animal Breeding and Genetics Program, IRTA, Torre Marimon, Caldes de Montbui, Spain.

[2]Université Paris-Saclay, INRAE, AgroParisTech, GABI, Jouy-en-Josas, France.

[3]IFIP-Institut du porc and Alliance R&D, Le Rheu, France.

[4]Hendrix Genetics Research Technology & Services B.V., Boxmeer, the Netherlands.

[5]Hypor B.V., Boxmeer, the Netherlands.

*Daniel Crespo-Piazuelo: daniel.crespo@irta.cat

Hervé Acloque: herve.acloque@inrae.fr

Olga González-Rodríguez: olga.gonzalez@irta.cat

Mayrone Mongellaz: mayrone.mongellaz@inra.fr

Marie-José Mercat: marie-jose.mercat@ifip.asso.fr

Marco C.A.M. Bink: Marco.Bink@hendrix-genetics.com

Abe E. Huisman: Abe.Huisman@hendrix-genetics.com

Yuliaxis Ramayo-Caldas: yuliaxis.ramayo@irta.cat

Juan Pablo Sánchez: JuanPablo.Sanchez@irta.cat

*Maria Ballester: maria.ballester@irta.cat

*Corresponding authors.

Daniel Crespo-Piazuelo [0000-0001-7896-2507];

Hervé Acloque [0000-0003-4761-1055];

Marie-José Mercat [0000-0002-3087-082X];

Marco C A M Bink [0000-0002-1278-2092];

Abe E Huisman [0000-0001-6572-1157];

Yuliaxis Ramayo-Caldas [0000-0002-8142-0159];

Juan Pablo Sánchez [0000-0001-8639-6146];

Maria Ballester [0000-0002-5413-4640]

Abstract

Background

In humans and livestock species, genome-wide association studies (GWAS) have been applied to study the association between variants distributed across the genome and a phenotype of interest. To discover genetic polymorphisms affecting the duodenum, liver, and muscle transcriptomes of 300 pigs from three different breeds (Duroc, Landrace and Large White), we performed expression GWAS between 25,315,878 polymorphisms and the expression of 13,891 genes in duodenum, 12,748 genes in liver, and 11,617 genes in muscle.

Results

More than 9.68×10^{11} association tests were performed, yielding 14,096,080 significantly associated variants, which were grouped in 26,414 expression quantitative trait locus (eQTL) regions. Over 56% of the variants were within 1Mb of their associated gene. In addition to the 100kb region upstream of the transcription start site, we identified the importance of the 100kb region downstream of the 3’UTR for gene regulation, as most

of the *cis*-regulatory variants were located within these two regions. We also observed 39,874 hotspot regulatory polymorphisms associated with the expression of 10 or more genes that could modify the protein structure or the expression of a regulator gene. In addition, two motifs (5’-GATCCNGYGTTGCYG-3’ and a poly(A) sequence) were enriched across the three tissues within the neighbouring sequences of the most significant SNPs in each *cis*-eQTL region.

Conclusions

The 14 million significant associations obtained in this study are publicly available and have enabled the identification of expression-associated *cis*-, *trans*- and hotspot regulatory variants within and across tissues, thus shedding light on the molecular mechanisms of regulatory variations that shape end-trait phenotypes.

Keywords

eQTL, hotspot, pig, RNA-Seq, WGS.

Background

Over the last decade, genome-wide association studies (GWAS) have been applied to study the association between genetic variants distributed across the genome of a species and traits of interest. Whether these traits are related to disease in humans or to production or health in livestock species, GWAS have shown that more than 88% of phenotype-associated variants are located outside protein-coding regions and are enriched in gene regulatory regions [1,2]. These noncoding variants may affect traits of interest by acting on gene regulation mechanisms, for example by affecting gene expression [3]. In this sense, gene expression can be considered as an “intermediate phenotype”, as it is expected to be more closely linked to genetic variations than conventional phenotypes [4]. Genetic variants that are significantly associated with the expression of a gene are called expression quantitative trait loci (eQTLs), and they are

commonly identified through expression GWAS (eGWAS), in which the expression of each gene is considered as a trait to pinpoint genomic regions involved in its regulation. If the distance of an eQTL relative to its associated gene is less than 1Mb, it is usually classified as a *cis*-eQTL [5]. Conversely, eQTLs located farther than 1Mb from their associated genes, or on another chromosome, are referred to as *trans*-eQTLs.

In pigs, previous eQTL studies have been performed in a breed-specific context using either low-density genotyping arrays or gene expression arrays [6–9]. Nowadays, eGWAS can be conducted with the comprehensive set of polymorphisms that segregate in a population thanks to the use of whole genome sequencing (WGS) data and genotype imputation. Such a strategy has the potential to reveal causal mutations responsible for the variation in gene expression levels. In humans, eGWAS have helped to detect the causal mutation for rare diseases, but only few studies have specifically aimed to uncover causal mutations associated with the transcriptomic variation of more than a particular set of genes [10]. Whole genome datasets are composed of millions of variants, thus increasing their complexity and the computing power required to analyse them. Furthermore, transcriptome datasets can include measures for tens of thousands of genes, further complicating the interpretation of results. For this reason, the majority of studies using both types of datasets have focused on identifying *cis*-eQTL regions associated with genes of interest, disregarding or analysing to a lesser extent *trans*-eQTL regions due to their complexity, small effect size, demanding computing requirements, and indirect regulation mechanisms [4,5,11,12]. Regions upstream from the TSS (transcription start site) have received particular attention due to their potential role as promoter or enhancer regions. Nevertheless, other downstream regulatory regions should not be ignored.

Apart from human and mouse, few studies have analysed how regulatory elements impact the phenotypes of other species [13]. To provide insight into the regulation mechanisms of gene expression in multiple tissues of livestock species, consortia such

as FAANG (Functional Annotation of ANimal Genomes) and FarmGTEx (Farm Animal Genotype-Tissue Expression) were recently established [14,15]. In this context, the GENE-SWitCH project [16] aims at characterising the functional elements of chicken and pig genomes, and describing genetic and epigenetic determinants of complex traits. Altogether, the functional annotation of genomes is expected to help advance genomic selection for the breeding industry towards more sustainable production systems. In addition, the identification of the regulatory regions responsible for the changes in gene expression may be translatable to other species. For example, pig biomedical models have played an important role for studying human diseases not only due to the similarity between both species in anatomical structure, genome, immunology, and physiology [17,18], but also due to their similar gene expression profiles [19–21].

Our study was developed in the framework of the GENE-SWitCH project with the objective of discovering genetic polymorphisms associated with the variation of gene expression levels in the duodenum, liver, and muscle of pigs. In particular, polymorphisms were studied regarding the proximity to their associated gene, as well as their potential role as hotspot regions or causal mutations.

Results

Whole genome and RNA sequencing

In this work we sequenced the whole genome of 300 pigs from three commercial pig populations (Duroc, Landrace, and Large White), which resulted in 44,127,400 genetic variants. After the filtering steps on minor allele frequency (MAF) and percentage missing, 25,315,878 polymorphisms remained for the association analyses. Variants were primarily classified as SNPs (74.9%), and to a lesser extent insertions (13.9%) or deletions (11.2%). In addition, the transition/transversion (Ts/Tv) ratio was 2.01.

In parallel, the transcriptomes of duodenum, liver, and skeletal muscle of the 300 pigs were sequenced. From the total sequenced reads, 92.1% were mapped against the pig reference genome. Out of these, 93.3% of the sequences were located inside gene regions (80.0% in exonic regions and 13.3% in intronic regions). After normalisation, filtering and quality control of the three transcriptomic datasets, 13,891 genes were found to be expressed in duodenum, 12,748 in liver, and 11,617 in muscle. The number of genes that were expressed in all three tissues was 10,719, while 14,916 genes were expressed in total among the three tissues (Fig. 1A).

Expression genome-wide association studies (eGWAS)

Among the three tissues, the eGWAS reported 14,096,080 significant associations (adjusted *p*-value≤0.05) with 10,019 different genes (Table 1). The full list of significant associations found across tissues can be accessed on the "Availability of data and materials" section. The number of expressed genes that had at least one significantly associated variant were 3,064 in duodenum (22.1%), 6,102 in liver (47.9%), and 5,559 in muscle (47.9%) (Fig. 1B). Out of these genes with any significant association, 863 were expressed simultaneously in the three tissues. Regarding the number of associated variants shared between tissues, 3.1% of the variants were associated with the same gene in duodenum, liver and muscle (Fig. 1C). Muscle was the tissue with the greatest number of unique associations, but duodenum and liver shared the greatest number of associated variants. Similar results were observed for the number of *cis*-regulatory variants shared across tissues, where 3.2% of the *cis*-regulatory variants were found in common (Fig. 1D).

Table 1. Number of significantly associated variants and eQTL regions per tissue.

Tissue	Significantly associated variants		eQTL regions*	
	No.	*cis*-regulatory variants (and %†)	No.	*cis*-eQTL regions (and %†)
Duodenum	3,162,603	1,650,766 (52.2%)	4,645	1,311 (28.2%)
Liver	4,340,467	2,436,245 (56.1%)	11,232	1,898 (16.9%)
Muscle	6,593,010	3,823,132 (58.0%)	10,537	2,604 (24.7%)
Total	14,096,080	7,910,143 (56.1%)	26,414	5,813 (22.0%)

*Reported only those eQTL regions that include at least two significantly associated variants.
†Percentage of the total No. found in tissue.

Out of the 14,096,080 significant associations, 76.72% were classified as SNPs, 13.72% as insertions and 9.56% as deletions. In total, 5,925,721 variants (23.4% of the 25M) were associated with at least one gene in one tissue. Out of these, 29.5% were novel variants, as they were not described in the Ensembl database (Release 106: April 2022).

For each gene and within each tissue, an eQTL region was defined at ±1Mb from any significantly associated polymorphism, merging them if they intersected. We considered for further analyses only the 26,414 eQTL regions that included at least two significant polymorphisms (Table 1). If we included the regions that were constituted by one single significant polymorphism, a total of 9,825, 28,429, and 23,409 eQTL regions would have been defined for duodenum, liver, and muscle, respectively. These 39.51-47.28% of eQTL regions constituted by a single significant polymorphism are indicative of putative spurious association signals, since linkage disequilibrium around a given location is expected to lead to a set of multiple significant signals.

Distribution of cis- and trans-regulatory variants with respect to their eQTL and gene region

On average, 56.1% of the significantly associated variants were located at less than 1Mb from their associated gene (i.e., *cis*-regulatory variants). However, this proportion was

much lower regarding *cis*-eQTL regions (22.0%), indicating that *cis*-eQTL regions comprised more significant polymorphisms, but *trans*-eQTL regions were more abundant.

To evaluate the distribution of significant polymorphisms on each eQTL region, their distance to the top polymorphism (i.e., the smallest *p*-value) was plotted for each tissue (Fig. 2). The density plot showed that most of the polymorphisms were at less than 1Mb from the top polymorphism of their eQTL region. Therefore, the use of a window of ±1Mb from the gene region to define a *cis*-regulatory variant seemed appropriate, as the linkage disequilibrium between a variant and the most significant polymorphism of its eQTL region rarely surpassed this 2Mb window.

We then assessed the distribution of the defined *cis*-regulatory variants within a 1Mb window prior to the start and posterior to the end sites of the studied genes (i.e., within the gene region +1Mb on both sides). The same pattern of distribution was observed for the three tissues (Fig. 3). The regions with the greatest number of *cis*-regulatory variants were those located within 100kb upstream the TSS and downstream the 3'UTR (untranslated region), whereas the number of *cis*-regulatory variants found within the open reading frame (ORF) of the gene was much lower.

Unsurprisingly, the top polymorphisms found in *cis*-eQTL regions had lower *p*-values than those found in *trans*-eQTL regions (Fig. 4). On average, 56.1% of the associations found were *cis*-regulatory variants, but only 22% of the annotated eQTLs were in *cis*. Therefore, most of the *cis*-eQTL regions were formed by a greater number of associated polymorphisms in linkage disequilibrium, whereas *trans*-eQTL regions, although numerous, had a lesser number of associated polymorphisms. Regarding the number of top polymorphisms found in common, 53 variants were associated with the same gene across the three tissues. Out of these, there were 6 *cis*-regulatory variants (Table 2).

Table 2. Top *cis*-regulatory variants found in common between duodenum, liver, and muscle eQTL regions.

Associated gene		Top cis-regulatory variant			
EnsemblID	Gene Name	Chromosome	Position	Reference Allele	Alternative Allele
ENSSSCG00000005103	*DET1*	1	190,113,347	T	TC
ENSSSCG00000013039	*NUDT22*	2	7,886,878	TC	T
ENSSSCG00000001398	*SLA-7*	7	24,180,079	C	CT
ENSSSCG00000011121	*CELF2*	10	60,516,892	C	A
ENSSSCG00000039915	*R3HCC1*	14	7,427,081	A	G
ENSSSCG00000028523	*HUS1*	18	48,525,415	T	C

Hotspot and top-hotspot regulatory polymorphisms, predicted consequences on protein structure and Gene Ontology analyses

A total of 5,183 hotspots (i.e., significant polymorphisms associated with the expression of 10 or more genes) were found in duodenum, 7,186 in liver, and 27,505 in muscle. Out of these, 110 hotspots in duodenum were predicted to have a moderate or high impact on the protein sequence, 133 in liver, and 452 in muscle (Supplementary Table S1). Besides, 102 of these hotspots were found in common across the three tissues and were located in 48 genes. Out of these, only 20 genes were simultaneously expressed on the three tissues, including 11 transcription factors and cofactors (AURKAIP1, HES4, NOC2L, TRIM28, ZNF134, ZNF274, ZNF544, and other 4 zinc finger proteins). Remarkably, these 102 hotspots shared among the three tissues were located within the same genomic region (56.3-64.5 Mb) on SSC6.

Through the joint analysis of hotspots and eQTL regions, top-hotspots can be defined as the most significantly associated polymorphism in at least 10 eQTL regions (Fig. 5A). Thus, 94, 176 and 84 out of the total hotspots previously defined for duodenum, liver, and muscle were declared as top-hotspots, respectively. In total, 23 top-hotspots were the top *cis*-regulatory variant of 11 genes among the three tissues (Fig. 5B, Table 3, Supplementary Table S2). Due to being simultaneously a top-hotspot and the most

significant polymorphism of its *cis*-eQTL region, they had a strong likelihood of being the causal mutations with a potential role as regulatory variants. Note however, that 22 additional top-hotspots, across tissues, were *cis*-regulatory variants of another 11 genes without being the most significantly associated polymorphism of its *cis*-eQTL region (Fig. 5C, Supplementary Table S2). Altogether, 22 genes with 45 top *cis*-regulatory hotspots were postulated as regulators among the three tissues. Remarkably, some of these were already described as transcription factors and cofactors: ARL2BP, CHD7, CHD8, LHX6, and ZNF331 in liver; and NFYC in muscle (Supplementary Table S2). By definition, and excluding those variants in complete linkage disequilibrium, some regulators had more than one top *cis*-regulatory hotspot, which were associated with the expression of a different number of genes.

Table 3. Top-hotspots that were also the most significant polymorphisms of their *cis*-eQTL region.

Tissue	EnsemblID	Gene Name	Top *cis*-regulatory hotspot	No. of co-expressed genes
duodenum	ENSSSCG00000006561	*SLC39A1*	4:96172709_C/CT	39
liver	ENSSSCG00000005530	*LHX6*	1:262892119_T/C	20
liver	ENSSSCG00000023140	*EIF2B4*	3:112449895_C/CT*	27
liver	ENSSSCG00000006231	*CHD7*	4:72782568_C/CTTT	35
liver	ENSSSCG00000035772	*CDH5*	6:26224686_CT/C	15
liver	ENSSSCG00000031793	*ZNF331*	6:56388054_T/C*	21
liver	ENSSSCG00000003680	*RALBP1*	6:98862179_A/G; 6:98862186_A/G; 6:98862192_T/A; 6:98862193_T/G; 6:98862199_CT/C; 6:98862206_G/A; 6:98862214_C/T; 6:98862215_C/T	86
liver	ENSSSCG00000002127	*CHD8*	7:77040284_A/AA	92
liver	ENSSSCG00000031538	*RNASE4*	7:78212187_G/GTGTGTGTA	36
liver	ENSSSCG00000038622	*HS3ST3A1*	12:56976226_C/CCAAAAAAAAA	50
muscle	ENSSSCG00000039550	*EMC4*	7:79160941_GA/G; 7:79246475_A/G; 7:79246477_G/A; 7:79561343_T/C; 7:79816434_T/G; 7:79816437_G/GT	324

*Only significant polymorphism of its cis-eQTL region.

In order to evaluate the modulation of the expression of several genes through changes in the expression of a regulator gene, pathway, gene ontology and co-expression analyses were carried out between the 22 genes postulated as regulators (i.e., with a top *cis*-regulatory hotspot) and the rest of the genes associated with the same regulatory variant in *trans*, henceforth referred as *trans*-associated-genes.

Among the 22 regulator genes, 1 was a long non-coding RNA (lncRNA) and another 2 were novel genes with no known ortholog in humans. All the remaining 19 genes participated together with their *trans*-associated-genes in at least one pathway. In general, most of the regulators were co-expressed with all their *trans*-associated-genes. The data on biological functions and pathways, and co-expression results for the 22 genes with top *cis*-regulatory hotspots are available in Supplementary Tables S3 and S4, respectively.

In duodenum, *SLC39A1* was the only putative regulator found, and it shared its role in transport and multicellular organism development pathways with 12 of its *trans*-associated-genes. Remarkably, *SLC39A1* showed co-expression with all its 38 *trans*-associated-genes, disregarding if they shared the same pathway.

The tissue with the greatest number of putative regulators was liver (14 genes). After the gene ontology analyses, the most relevant regulators based on the shared functions with their *trans*-associated-genes were *CHD7*, *CHD8*, *CTSC*, and *RALBP1*. CHD7 and CHD8 are chromodomain helicase DNA binding proteins, which were involved in gene expression together with other 19 and 27 *trans*-associated-genes, respectively. Furthermore, they also participate in chromosome organization, and animal organ and embryo development. In addition, *CHD7* was also involved in growth regulation together with other 3 *trans*-associated-genes. Despite the *cis*-polymorphism of *CHD8* being associated with 92 genes, *CHD8* was only co-expressed with 36% of them, while *CHD7* was co-expressed with all its 34 *trans*-associated-genes. The *CTSC* gene was a regulator of multicellular organismal development along with other 16 *trans*-associated-

genes, but it also participated in the regulation of the immune system process and in the response to organic substances. Intriguingly, *CTSC* was only co-expressed with 8 out of its 49 *trans*-associated-genes. Participating in a plethora of different functions, RALBP1 was a protein involved in the regulation of metabolic process together with other 38 *trans*-associated-genes, but it was also involved in phosphorylation, mitochondrion organization, and the regulation of GTPase activity and developmental processes. It is also worth noting that *RALBP1* showed co-expression with all of its 85 *trans*-associated-genes.

Out of the 4 regulators in muscle, *EMC4* was the gene with the greatest number of *trans*-associated-genes. Out of its 323 *trans*-associated-genes, *EMC4* was co-expressed with 314 (97%) of them. Among the pathways shared with 44 and 69 of them, *EMC4* participated in the apoptotic process and in organelle organization, respectively. *GCAT* was involved with other 29 genes in the metabolism of amino acids and derivatives, and it was co-expressed with 37 out of its 38 *trans*-associated-genes. Although *NFYC* was only the potential regulator of other 10 *trans*-associated-genes, it was co-expressed with 7 of them, and together with all but one of its *trans*-associated-genes, was implicated in the cellular nitrogen compound metabolic process, similar to the aforementioned metabolism of amino acids. In addition, as a transcription factor, NFYC and 2 other *trans*-associated-genes participated in the regulation of transcription by RNA polymerase II. In this context, *POLR2F*, the remaining regulator, encodes a subunit of RNA polymerase II, and thus played a role in gene expression together with 28 other *trans*-associated-genes. *POLR2F* was also found in pathways related with cellular nitrogen compound biosynthetic process, developmental biology and immunity, but did not show a great percentage of co-expression with its *trans*-associated-genes (16% and 67%, depending on the top *cis*-regulatory hotspot considered).

Motifs in cis-regulatory SNPs

Regions of 20bp (base pairs) around the top SNPs in *cis*-eQTL regions (852 SNPs in duodenum, 1,142 SNPs in liver, and 1,605 SNPs in muscle) were selected to perform motif discovery using the MEME Suite. Two motifs were recurrently found in the three tissues (Fig. 6).

The first motif was composed of a poly(A) sequence, or a poly(T) sequence if the reverse complement sequence was considered. This motif was found in 44 genes in duodenum, 79 in liver, and 103 in muscle (Supplementary Table S5).

The second motif was 5'-GATCCNGYGTTGCYG-3', which was found in 22 genes in duodenum, 21 in liver, and 36 in muscle (Supplementary Table S5). Remarkably, a guanine or a cytosine was almost always found on the mutation site in the reference or the alternative sequence.

Discussion

In the present study, we provide a catalogue of eQTLs associated with the expression levels of local and distal transcripts in liver, duodenum, and muscle tissues. More than 9.68×10^{11} combinations between 25M polymorphisms and 14,916 genes expressed in duodenum, liver, and muscle were tested following an eGWAS approach, resulting in 14,096,080 significant associations. After filtering out eQTL regions that contained a single associated polymorphism, the remaining significant associations were grouped into 26,414 eQTL regions.

Among these significant associations, only 443,857 polymorphisms (3.1%) were associated with the same gene across the three tissues, reflecting a high proportion of identified genetic variants with tissue-specific regulatory potential. This is in agreement with what has been observed in humans, as common regulatory variants are less abundant than tissue-specific variants, which are usually *trans*-regulatory variants [22].

However, although *cis*-regulatory variants are usually found in common across tissues with a lesser tissue-specificity than *trans*-regulatory variants, we observed a similar ratio (3.2%) of shared *cis*-regulatory variants across the three tissues, which may be due to the limited relationship between the three tissues. Among the three analysed tissues, muscle was the tissue with the greatest number of significant associations, followed by liver and duodenum. This difference in significant associations between muscle and the other two tissues may be due to the increased selective pressure that muscle has experienced over the last century, as lean meat percentage was one of the main traits considered in breeding programs [23]. Nowadays, pig breeds highly differ in muscle growth and structure [24,25], which are in turn influenced by pre- and post-natal muscle cell expression [26,27]. Although moderate overlapping of eQTLs between muscle and liver has already been reported in pigs [9], we observed the greatest number of shared significant associations between duodenum and liver (1,121,266). This may be due to the shared embryonic origin of the duodenum and liver tissues, which originate from the endodermal layer, whereas muscle has its origin in the mesodermal layer. Similar results were also reported in humans, where a strong correlation was observed between closely related tissues and shared eQTLs [22].

Since the locations of regulatory elements are not well-defined for most genes, the definition of a window size for annotating eQTL regions varies across studies, from 10kb in yeast to 20Mb in mice [28]. Traditionally, most of the literature defines *cis*-eQTL regions as 1Mb window from the TSS of the target gene [4,5,7,29,30]. However, *cis*-regulatory variants can be located further than 1Mb from the TSS [31]. In addition, *cis*-regulatory variants can also be found within the 3’UTR of genes [32], participating in the post-transcriptional regulation of gene expression by altering the binding sites of RNA molecules such as microRNAs or lncRNAs, among other mechanisms. Thus, in this study, we defined a *cis*-regulatory variant if it was located within the expanded gene region (i.e., the gene region ±1Mb). In accordance with some studies that reported that

most of the *cis*-regulatory variants were located within 100kb from the TSS [15,33], we observed the same pattern across duodenum, liver and muscle. Nonetheless, the *cis*-regulatory variants located within 100kb from the 3'UTR were as abundant as those located within 100kb upstream the TSS, which implies that studies on regulatory variants should not be focused on the TSS neighbouring region alone. As expected, the number of *cis*-regulatory variants located within ORFs was much lower than those located within 100kb from the TSS and the 3'UTR.

In addition, as eQTL regions were annotated by intersecting the expression-associated variants that were located at less than 2 Mb, the most significant polymorphism of a *cis*-eQTL region could be located further than 1Mb from its associated gene. The consideration of this 1Mb window is supported by the observed linkage disequilibrium between an associated variant and the most significant polymorphism within an eQTL region, which rarely surpassed 2Mb. Nonetheless, the definition of *cis*- and *trans*-eQTLs cannot be solely based on the distance to the associated gene, as this may change depending on the structure of the population used and the linkage disequilibrium between the variants under consideration. For example, the widest (60Mb) eQTL region was located on SSCX, as the recombination rate in heterosomes is much lower than in autosomes.

Although the number of *cis*-eQTLs regions was lower than the number of *trans*-eQTL regions, *cis*-eQTL regions had a greater number of associated polymorphisms and their most significant polymorphisms usually had lower *p*-values. Previous studies in humans have already described weaker and more indirect effects of *trans*-regulatory elements on gene expression [4,33]. Moreover, due to the multiple testing correction that must be applied, *trans*-eQTL regions with small effects are particularly difficult to detect [34]. However, defining hotspot regions can help investigate and determine the effect of *trans*-eQTL regions on gene regulation, as it avoids spurious associations and increases detection power.

Despite having the lowest number of annotated eQTL regions among the three tissues, the duodenum had the greatest number of annotated *cis*-eQTL regions. On the contrary, the liver had the greatest amount of annotated eQTL regions, but the lowest percentage of *cis*-eQTL regions. This may be indicative of the complexity of the regulatory mechanisms in liver, which would be more dependent on *trans*-regulatory elements. Following this hypothesis, the amount of hotspot *cis*-regulatory variants was greatest in liver, whereas a single hotspot *cis*-regulatory variant was found in duodenum.

As previously mentioned, 3.2% of the *cis*-regulatory variants were shared between duodenum, liver and muscle, and only 6 top *cis*-regulatory variants were found in common among the three tissues. In agreement with our findings, the existence of common regulatory elements across tissues has already been documented [5,30,35,36], including shared *cis*-eQTLs between *gluteus medius* muscle and liver in a Duroc population [9]. Although the 6 *cis*-regulatory variants found in our study were the most significantly associated signal of their *cis*-eQTL regions, indicating a great potential to be the causal mutations and explain the variability in expression of their associated genes, other polymorphisms in linkage disequilibrium with them should not be discarded.

Through the modification of expression levels of their associated genes, the 6 top *cis*-regulatory variants found across the three tissues have the potential to determine production and health traits in pigs. *CELF2* was suggested as a candidate gene for backfat thickness in Large White pigs [37]. *HUS1* and *NUDT22* were related to the intramuscular fat content in pigs [38,39]. In addition, *HUS1* was also proposed as a candidate gene for meat colour in muscle from Duroc × Luchuan pigs [7] and for the abundance of alanine aminotransferase in blood from Large White pigs [40]. After an *in vitro* infection with *Salmonella*, *R3HCC1* was downregulated in porcine neutrophils [41]. In blood from the same Large White pig population as that used in this study, a *cis*-regulatory variant was also associated with the expression of *SLA-7* [8]. The *cis*-regulatory variant (rs80859275) was located at ~478kb from the most significant signal

found in our data across the three tissues. Thus, it could be in linkage disequilibrium with the potential causal mutation reported by our study and extend its regulatory role to four tissues (blood, duodenum, liver, and muscle). The importance of *SLA-7* lies in its participation on the porcine major histocompatibility complex, swine leukocyte antigen (SLA), although its exact functions remain to be determined [42].

Among the putative causal mutations on hotspot *trans*-regulators (i.e., polymorphisms on the coding region of a gene that were significantly associated with the expression of more than 10 genes in *trans*), 3 missense mutations associated with the expression of 33 genes in *trans* were found on the coding region of the *NOC2L* gene. NOC2L is a transcription factor which inhibits the histone acetyltransferase activity and prevents all core histones from being acetylated [43]. On the coding region of another gene, *TRIM28* had 2 predicted missense mutations that were associated with a total of 29 genes in *trans*. This transcriptional regulator acts as a repressor of gene expression by recruiting CHD3 [44]. TRIM28 also participates in host innate immune response [45] and negatively regulates aggresome formation [46], among several other functions. In pigs, *TRIM28* knockdown in gestating sows produced epigenetic variations in their embryos, including those of the promoter region of the *IGF2* gene, affecting their developmental processes [47].

Based on the genes with top *cis*-regulatory hotspots, 22 were postulated as regulators. Out of this list, it is worth mentioning that *SLC39A1*, the only regulator found on duodenum and co-expressed with all of its *trans*-associated-genes, was not previously described as a transcription factor or cofactor in the literature, but rather as a regulator of zinc homeostasis in the gut [48]. Both CHD7 and CHD8 are members of the chromodomain-helicase-DNA binding protein family and participate in transcription regulation by chromatin remodelling [49]. However, their interpretation as liver-specific transcriptional regulators in our study is obscured, as both *CHD7* and *CHD8* are ubiquitously expressed, but no top *cis*-regulatory hotspots were described for them in the

other two tissues of our study. Nonetheless, human patients with a lower expression of *CHD8* have a favourable prognosis in liver cancer [50]. Apart from participating in immunity, the expression of *CTSC* was also associated with feed efficiency and loin tenderness in pigs [51,52]. In muscle, NFYC was the only regulator previously described as a transcription factor. NFYC binds to 5'-CCAAT-3' motifs and participates in muscle cell differentiation [53]. In pigs, *NFYC* was reported as co-associated with lean percentage and average daily gain in a regulatory gene network [54]. The gene with the greatest number of co-expressed genes was *EMC4*. Despite *EMC4* being expressed in the three tissues, its hotspot regulatory role was only observed in muscle. The protein encoded by this gene is mainly located in the endoplasmic reticulum membrane and participates in the development of muscle [55,56].

We found across the three tissues, the same two motifs using the sequences surrounding the most significant SNPs in *cis*-eQTLs. The first of them, a poly(A) motif, was a short tandem repeat (STR) of adenines that could also include few other bases. Adenine-rich STRs are the most common sequences found in the human genome [57] and their potential role as transcriptional regulators may be due to a variety of reasons. For example, some of the sequences were similar to the 5'-AAUAAA-3' endonucleolytic cleavage site prior the addition of the poly(A) tail [58]. However, not all of the significantly associated polymorphisms were located in the 3′ flanking region. Hence, other mechanisms in which poly(A) or poly(T) could affect the expression of large RNA molecules were the T-loop RNA folding motif [59], and nucleosome control and accessibility [60,61]. The second motif, 5'-GATCCNGYGTTGCYG-3', was usually found in the 5′ flanking region of the associated genes. Its potential regulatory role was also supported by the fact that the mutated base in the reference or the alternative sequence was almost always a guanine or a cytosine, and thus possibly impairing a CpG site. In agreement with its potential role as regulator, the same motif was found in the promoter

regions of human genes, despite the fact that no transcription factor was found to bind on such sequences.

Conclusions

In conclusion, we have reported more than 14 million significant associations between 5,925,721 variants and the expression of 10,019 genes in duodenum, muscle and liver, which are publicly available. We have also reported that most of the *cis*-regulatory variants were equally abundant within the 100kb upstream the TSS or 100kb downstream the 3'UTR. In addition, our results have allowed the identification of genomic *cis*, *trans* and hotspot regions associated with the expression of such genes within and across tissue, which will shed light on the molecular mechanisms of regulatory variations that shape end-trait phenotypes.

Material and Methods

Ethics statement

Since the pigs analysed in this study were not subjected to any experimental procedures given that samples were taken *post-mortem*, the study was exempt from the European Union Directive 2010/63/EU about the protection of animals used in experimentation. Duroc and Landrace pigs were reared and slaughtered in a commercial farm and abattoir following Spanish national and institutional guidelines for Good Experimental Practices. Large White pigs were reared and slaughtered according to procedures approved by the French Veterinary Services at INRAE UE3P France Génétique Porc phenotyping facilities (user establishment agreement number C-35–240–7; UE3P [62]).

Animal Material

A total of 300 pigs of three different breeds (n=100 Duroc, n=100 Landrace and n=100 Large White) were used in this study. Duroc animals were distributed in three batches balancing gender, 50 males and 50 females, and belonged to 33 litters obtained from 33 sows and 10 boars. Large-White pigs were distributed in 4 batches of uncastrated males and belonged to 84 litters obtained from 84 sows and 43 boars. Landrace animals were taken from one batch, 39 males and 61 females, and belonged to 74 litters obtained from 74 sows and 18 boars. Each breed was raised in a different farm and fed *ad libitum* with a commercial cereal-based diet. Pigs were slaughtered at 5-7 months of age in a commercial abattoir and blood, duodenum, liver, and muscle samples were collected. Full information on the animal material is publicly available on the FAANG data portal (accession number requested).

DNA and RNA extraction and sequencing

Genomic DNA was extracted using NucleoSpin Blood kit (Macherey-Nagel, Düren, Germany) on blood samples from Duroc and Landrace pigs, and on liver samples from Large White pigs using QIAamp DNA Mini Kit (Qiagen, Hilden, Germany). Duodenum, liver, and muscle samples were homogenised using biodisruptor and bead tubes (Lysing matrix D). Duodenum and liver RNA was extracted using a chemagic™ 360 instrument with RNA Tissue10 Kit H96 (PerkinElmer, Baesweiler, Germany). Muscle RNA was extracted using RiboPure™ RNA Purification Kit (Invitrogen, Carlsbad, CA, USA) and RNeasy Fibrous Tissue Mini Kit (Qiagen, Hilden, Germany). Detailed protocols are publicly available on the FAANG data portal [63,64]. DNA and RNA were quantified in a NanoDrop ND-1000 spectrophotometer (NanoDrop Technologies; Wilmington, DE, USA). Purity and integrity of RNA was assessed in a Bioanalyzer-2100 (Agilent Technologies, Santa Clara, CA, USA). RIN values ranged from 6 to 9.2 for duodenum and liver samples, while muscle samples had RIN>8 values. For sequencing, more than

2 μg of total RNA in a concentration range of 50-200 ng/μl was provided. Libraries were prepared using the TruSeq Stranded mRNA Sample Preparation kit (Illumina, San Diego, Ca, USA). WGS libraries were constructed with an insert size of 470 bp, whereas the insert size of transcriptomic libraries was 285 bp. All DNA samples (n=300) were sequenced with a yield >30Gb, resulting in a WGS depth of about 10X. For RNA-seq, duodenum, liver, and muscle samples (n=900) were sequenced with a depth of >90M reads. All samples were paired-end sequenced (2 × 150 bp) in an Illumina NovaSeq6000 platform (RRID:SCR_020150) at *Centro Nacional de Análisis Genómico* (CNAG-CRG; Barcelona, Spain).

Mapping and annotation of DNA and RNA reads

Quality of raw DNA and RNA sequenced reads was assessed with the FastQC (RRID:SCR_014583) software [65]. DNA sequences were mapped against the reference genome (*Sscrofa11.1* assembly) with BWA-MEM/0.7.17 [66]. Alignment files containing only properly paired, uniquely mapping reads without duplicates were processed using Picard (RRID:SCR_006525) to add read groups and to remove duplicates.

Genetic variant calling was conducted with GATK (RRID:SCR_001876) /4.1.8.0 HaplotypeCaller [67] to extract SNPs and indels from whole genome sequences. HaplotypeCaller was used with the default parameters with the exception of setting the "--minimum-mapping-quality" to 20. Then, joint genotyping was carried out with GATK/4.1.8.0 CombineGVCFs to obtain a multi-sample gVCF file. Thereafter, BCFtools (RRID:SCR_005227) /1.9 norm [68] was used to split multiallelic sites (SNPs and indels) into multiple rows. For downstream analyses, genetic variants were filtered if the minor allele frequency was below 5% and/or if there was more than 10% missing genotype data using PLINK (RRID:SCR_001757) /v1.90b3.42 [69].

RNA sequences were mapped against the reference genome (*Sscrofa11.1* assembly) and the Ensembl Genes 101 annotation database with STAR (RRID:SCR_004463) /v2.5.3a [70], and counts were quantified with RSEM (RRID:SCR_013027) /1.3.0 [71]. During the filtering step, lowly expressed genes (counts per million (cpm)<10/minimum library size in millions), and those missing in more than 20% of the animals, were removed. Then, within-tissue counts were normalised by TMM (trimmed mean of M-values) and transformed to cpm using $\log_2$ and a prior count of 1 with the cpm function of the edgeR (RRID:SCR_012802) /3.30.3 Bioconductor package [72]. In addition, to avoid normalisation artefacts, raw counts with a value of 0 were replaced by NA.

The distribution of each normalised dataset was assessed by applying the Shapiro-Wilk test to each expressed gene following a leave-one-out procedure, i.e., taking out a sample and conducting the normality tests in the remaining 299 samples. Two samples from duodenum and three from muscle significantly reduced the number of genes with approximately normally distributed expressions values and were thus considered outliers and removed from the analyses.

Expression genome-wide association studies (eGWAS)

For each of the three tissues, eGWAS were carried out between the filtered polymorphisms and the normalised expression data applying the following model with the fastGWA tool from GCTA/1.93.2 [73]:

$$y_{hijk} = sex_{hj} + breed_{hk} + u_{hi} + s_{il} \cdot a_{hl} + e_{hijk}$$

where y_{hijk} corresponds to the expression of the hth gene in the ith individual of sex j and belonging to the kth breed; sex_{hj} corresponds to the jth sex effect (two levels); $breed_{hk}$ corresponds to the kth breed effect (three levels); u_{hi} is the infinitesimal genetic effect of the individual i, with u~MVN(0,G·σ^2_u), where G is the genomic relationship matrix calculated using the filtered autosomal polymorphisms as described in [74] and σ^2_u is the

additive genetic variance to be estimated; s_{il} is the genotype (coded as 0, 1 or 2) for the lth polymorphism; and a_{hl} is the allele substitution effect of the lth polymorphism on the expression level of the hth gene; and finally, e_{hijk} is a residual error term. Then, Bonferroni correction was applied to calculate genome-wide significance thresholds using the p.adjust function from the stats/4.0.4 R base package. Only those associations with an adjusted *p*-value≤0.05 were considered significant.

eQTL clustering and consequence prediction

Preliminary eQTL regions were considered by clustering the significant polymorphisms at a distance of less than 2Mb from each other. To reduce the number of false positives, only eQTL regions with a minimum of two polymorphisms were retained. Then, eQTL regions were extended 1Mb on each side of the previously defined regions. Gene positions were extracted with the BioMart tool [75] from the Ensembl Genes 101 annotation database. Significant polymorphisms that were located at less than 1Mb from their associated gene were defined as *cis*-regulatory variants. Therefore, eQTL regions containing a *cis*-regulatory variant were considered *cis*-eQTL regions. The remaining regions were considered *trans*-eQTL regions.

Functional predictions of the significant polymorphisms were performed with the Variant Effect Predictor tool [76] on the Ensembl Genes 106 annotation database.

Hotspot and top-hotspot polymorphism definitions and network analysis

In the context of this study, a hotspot was defined as a polymorphism associated with the expression of at least 10 genes. Further, a top-hotspot was defined as any hotspot that was the most significantly associated polymorphism (smallest *p*-value) in at least 10 eQTL regions. Genes with associated top-hotspots were checked for transcription factors and cofactors in the AnimalTFDB (RRID:SCR_001624) /v3.0 [77]. Top-hotspot

regulatory polymorphisms that were classified as *cis*-regulatory variants were further studied through network analysis. For that purpose, we extracted all genes that were significantly associated with the same top-hotspot, including the regulator gene associated in *cis*, and identified over-represented gene ontology terms and KEGG pathways with the ClueGO plugin [78] from Cytoscape (RRID:SCR_003032) [79]. In addition, co-expression between each regulator and its *trans*-associated-genes was assessed with the Partial Correlation and Information Theory (PCIT) approach [80], a network-based approach that combines partial correlation coefficient with information theory to identify significant correlations between each possible combination of genes.

Motif discovery

As motif discovery in variable sequences is made difficult by the presence of elements such as indels, only SNPs that were top *cis*-regulatory variants were considered for the analysis. Two sequences ±10bp of the position of the *cis*-SNP were extracted from the reference genome (*Sscrofa11.1* assembly), one including the reference SNP and the other with the alternative SNP. For each tissue, the two sets of sequences with the reference and the alternative alleles were submitted together to the MEME Suite (RRID:SCR_001783) /5.4.1 web-tool [81] to perform motif discovery trough the MEME tool [82]. Default parameters were used, but the maximum number of motifs to be searched was set to 15. Then, relevant consensus motifs were scanned against the same dataset with the FIMO tool [83] to assess the number of occurrences in a given tissue. Only those hits with a q-value≤0.1 were considered significant.

Data availability

The raw sequence data that support the findings of this study have been deposited in the FAANG data portal with the BioProject accession codes PRJEB58030 and

PRJEB58031. The results of the eGWAS across tissues have been made publicly available on the following repository [84]. All supporting data and materials are available in the GigaScience GigaDB database [85].

Figure legends

Figure 1. Venn diagram representing the shared elements across duodenum, liver, and muscle.

(A) Number of expressed genes. (B) Number of expressed genes with at least one significantly associated variant. (C) Number of significantly associated variants. (D) Number of *cis*-regulatory variants.

Figure 2. Density plot (black curve) representing the distance between each significantly associated polymorphism to the peak.

The peak is defined as the most significant polymorphism of an eQTL region (or their mean, if multiple). The significance of the polymorphisms is provided in blue dots. Sexual and mitochondrial chromosomes have been excluded. (A) Duodenum. (B) Liver. (C) Muscle.

Figure 3. Distribution of *cis*-regulatory variants within their *cis*-eQTL region grouped by distance.

The distance has been calculated between the position of each *cis*-regulatory variant and the position of the proximal transcription start site (TSS) or the 3'UTR (untranslated region) of their associated gene, whichever is closest. Those cis-regulatory variants that were located within the open reading frame (ORF) were placed between the TSS and the 3'UTR. (A) Duodenum. (B) Liver. (C) Muscle.

Figure 4. Comparison between the significance values of the top polymorphisms of *cis*-eQTL and *trans*-eQTL regions.

Boxplots have been drawn for each tissue. Sexual and mitochondrial chromosomes have been excluded.

Figure 5. Definition of hotspot, top-hotspot and the two types of top *cis*-regulatory hotspots.

Any polymorphism significantly associated with the expression of a particular gene is marked as a black dot, whereas *cis*-regulatory variants are marked in green. A red dot represents the most significantly associated polymorphism within an eQTL region. (A) A hotspot is defined as any polymorphism significantly associated with the expression of 10 genes or more; within them, those hotspots that are the most significantly associated polymorphism of at least 10 eQTL regions are defined as top-hotspots. (B) Top *cis*-regulatory hotspots are top-hotspots that are the most significantly associated polymorphism of their *cis*-eQTL region. (C) The other type of top cis-regulatory hotspots are top-hotspots that are *cis*-regulatory variants (orange) but are not the most significant polymorphism of their *cis*-eQTL region.

Figure 6. Sequence logo for the consensus DNA motifs found in common in the three tissues.

Mutations were located in position 11 on the left motif and usually found in position 6 on the right motif.

Supplementary material

Supplementary Table S1. List of hotspot regulatory polymorphisms that could have a moderate or high impact on the protein structure and their associated genes.

Supplementary Table S2. List of hotspot regulatory polymorphisms that were *cis*-regulatory variants and their associated genes.

Supplementary Table S3. ClueGO results for each regulator gene with a top hotspot in *cis* that was associated with the expression of more than 10 genes in *trans*. Only the pathways where the regulator gene is present are included.

Supplementary Table S4. Co-expression results for the 22 genes with hotspot *cis*-regulatory variants.

Supplementary Table S5. FIMO results for the two motifs found and the distance between the mutation and the proximal TSS and 3'UTR of their associated gene.

Declarations

List of abbreviations

3'UTR: 3' untranslated region

bp: base-pair

cpm: counts per million

eGWAS: expression genome-wide association studies

eQTLs: expression quantitative trait loci

GWAS: genome-wide association studies

lncRNA: long non-coding RNA

MAF: minor allele frequency

ORF: open reading frame

PCIT: Partial Correlation and Information Theory

SLA: swine leukocyte antigen

STR: short tandem repeat

TMM: trimmed mean of M-values

Ts/Tv: transition/transversion ratio

TSS: transcription start site

WGS: whole genome sequencing

Consent for publication

Not applicable.

Competing interests

The authors declare that they have no competing interests.

Funding

This project is part of GENE-SWitCH (https://www.gene-switch.eu) and has received funding from the European Union's Horizon 2020 Research and Innovation Programme under the grant agreement n° 817998. It is also part of EuroFAANG (https://eurofaang.eu), a synergy of five Horizon 2020 projects that share the common goal to discover links between genotype to phenotype in farmed animals and meet global FAANG objectives. Y.R.-C. was financially supported by a Ramon y Cajal contract (RYC2019-027244-I) from the Spanish Ministry of Science, Innovation and Universities. Some of the authors belonged to a Consolidated Research Group AGAUR, ref. 2021-SGR-01552.

Authors' contributions

M.B. designed the study. M.B., M.-J.M., M.C.A.M.B. and A.E.H. supervised the generation of the animal material used in this work. H.A., O.G.-R., M.M., M.-J.M., Y.R.-C. and M.B. performed the sampling. H.A., O.G.-R., M.M. and M.B. performed the DNA and RNA extractions. D.C.-P. performed the bioinformatic analyses. Y.R.-C. performed the co-expression analysis. D.C.-P., Y.R.-C., J.P.S. and M.B. analysed the data and interpreted the results. D.C.-P. and M.B. wrote the manuscript. All authors read and approved the submitted version of the manuscript.

Acknowledgements

The authors would like to thank Dr. Andrea Rau for proofreading the manuscript.

References

1. Maurano MT, Humbert R, Rynes E, Thurman RE, Haugen E, Wang H, et al. Systematic localization of common disease-associated variation in regulatory DNA. Science. 2012;337: 1190–5. doi:10.1126/science.1222794

2. Hindorff LA, Sethupathy P, Junkins HA, Ramos EM, Mehta JP, Collins FS, et al. Potential etiologic and functional implications of genome-wide association loci for human diseases and traits. Proc Natl Acad Sci U S A. 2009;106: 9362–7. doi:10.1073/pnas.0903103106

3. Ward LD, Kellis M. Interpreting noncoding genetic variation in complex traits and human disease. Nat Biotechnol. 2012;30: 1095–106. doi:10.1038/nbt.2422

4. Stranger BE, Nica AC, Forrest MS, Dimas A, Bird CP, Beazley C, et al. Population genomics of human gene expression. Nat Genet. 2007;39: 1217–24. doi:10.1038/ng2142

5. GTEx Consortium. Human genomics. The Genotype-Tissue Expression (GTEx) pilot analysis: multitissue gene regulation in humans. Science. 2015;348: 648–60. doi:10.1126/science.1262110

6. Ballester M, Ramayo-Caldas Y, Revilla M, Corominas J, Castelló A, Estellé J, et al. Integration of liver gene co-expression networks and eGWAs analyses highlighted candidate regulators implicated in lipid metabolism in pigs. Sci Rep. 2017;7: 46539. doi:10.1038/srep46539

7. Liu Y, Liu X, Zheng Z, Ma T, Liu Y, Long H, et al. Genome-wide analysis of expression QTL (eQTL) and allele-specific expression (ASE) in pig muscle identifies candidate genes for meat quality traits. Genet Sel Evol. BioMed Central; 2020;52: 59. doi:10.1186/s12711-020-00579-x

8. Maroilley T, Lemonnier G, Lecardonnel J, Esquerré D, Ramayo-Caldas Y, Mercat MJ, et al. Deciphering the genetic regulation of peripheral blood transcriptome in pigs through expression genome-wide association study and allele-specific expression analysis. BMC Genomics. 2017;18: 967. doi:10.1186/s12864-017-4354-6

9. González-Prendes R, Mármol-Sánchez E, Quintanilla R, Castelló A, Zidi A, Ramayo-Caldas Y, et al. About the existence of common determinants of gene expression in the porcine liver and skeletal muscle. BMC Genomics. 2019;20: 518. doi:10.1186/s12864-019-5889-5

10. Brown AA, Viñuela A, Delaneau O, Spector TD, Small KS, Dermitzakis ET. Predicting causal variants affecting expression by using whole-genome sequencing and RNA-seq from multiple human tissues. Nat Genet. 2017;49: 1747–1751. doi:10.1038/ng.3979

11. Nica AC, Montgomery SB, Dimas AS, Stranger BE, Beazley C, Barroso I, et al. Candidate causal regulatory effects by integration of expression QTLs with

complex trait genetic associations. Gibson G, editor. PLoS Genet. 2010;6: e1000895. doi:10.1371/journal.pgen.1000895

12. Grundberg E, Small KS, Hedman ÅK, Nica AC, Buil A, Keildson S, et al. Mapping cis- and trans-regulatory effects across multiple tissues in twins. Nat Genet. 2012;44: 1084–9. doi:10.1038/ng.2394

13. Nguyen QH, Tellam RL, Naval-Sanchez M, Porto-Neto LR, Barendse W, Reverter A, et al. Mammalian genomic regulatory regions predicted by utilizing human genomics, transcriptomics, and epigenetics data. Gigascience. 2018;7: 1–17. doi:10.1093/gigascience/gix136

14. Andersson L, Archibald AL, Bottema CD, Brauning R, Burgess SC, Burt DW, et al. Coordinated international action to accelerate genome-to-phenome with FAANG, the Functional Annotation of Animal Genomes project. Genome Biol. 2015;16: 57. doi:10.1186/s13059-015-0622-4

15. Liu S, Gao Y, Canela-Xandri O, Wang S, Yu Y, Cai W, et al. A multi-tissue atlas of regulatory variants in cattle. Nat Genet. 2022;54: 1438–1447. doi:10.1038/s41588-022-01153-5

16. The GENE-SWitCH project webpage. Available: www.gene-switch.eu

17. Lunney JK, Van Goor A, Walker KE, Hailstock T, Franklin J, Dai C. Importance of the pig as a human biomedical model. Sci Transl Med. 2021;13: eabd5758. doi:10.1126/scitranslmed.abd5758

18. Hou N, Du X, Wu S. Advances in pig models of human diseases. Anim Model Exp Med. 2022;5: 141–152. doi:10.1002/ame2.12223

19. Klymiuk N, Blutke A, Graf A, Krause S, Burkhardt K, Wuensch A, et al. Dystrophin-deficient pigs provide new insights into the hierarchy of physiological derangements of dystrophic muscle. Hum Mol Genet. 2013;22: 4368–82.

doi:10.1093/hmg/ddt287

20. Li X, Tang XX, Vargas Buonfiglio LG, Comellas AP, Thornell IM, Ramachandran S, et al. Electrolyte transport properties in distal small airways from cystic fibrosis pigs with implications for host defense. Am J Physiol Lung Cell Mol Physiol. 2016;310: L670-9. doi:10.1152/ajplung.00422.2015

21. Flisikowska T, Stachowiak M, Xu H, Wagner A, Hernandez-Caceres A, Wurmser C, et al. Porcine familial adenomatous polyposis model enables systematic analysis of early events in adenoma progression. Sci Rep. 2017;7: 6613. doi:10.1038/s41598-017-06741-8

22. GTEx Consortium. Genetic effects on gene expression across human tissues. Nature. 2017;550: 204–213. doi:10.1038/nature24277

23. Merks JWM. One century of genetic changes in pigs and the future needs. BSAP Occas Publ. 2000;27: 8–19. doi:10.1017/S1463981500040498

24. Wood J, Whittemore CT. Pig meat and carcass quality. In: Kyriazakis I, Whittemore CT, editors. Whittemore’s Science and Practice of Pig Production. 3rd ed. Oxford, UK: Blackwell Publishing Ltd; 2006. doi:10.1002/9780470995624

25. Lee SH, Choe JH, Choi YM, Jung KC, Rhee MS, Hong KC, et al. The influence of pork quality traits and muscle fiber characteristics on the eating quality of pork from various breeds. Meat Sci. 2012;90: 284–91. doi:10.1016/j.meatsci.2011.07.012

26. Muráni E, Murániová M, Ponsuksili S, Schellander K, Wimmers K. Identification of genes differentially expressed during prenatal development of skeletal muscle in two pig breeds differing in muscularity. BMC Dev Biol. 2007;7: 109. doi:10.1186/1471-213X-7-109

27. Rehfeldt C, Fiedler I, Dietl G, Ender K. Myogenesis and postnatal skeletal muscle

cell growth as influenced by selection. Livest Prod Sci. 2000;66: 177–188. doi:10.1016/S0301-6226(00)00225-6

28. Williams RBH, Chan EKF, Cowley MJ, Little PFR. The influence of genetic variation on gene expression. Genome Res. 2007;17: 1707–16. doi:10.1101/gr.6981507

29. Fauman EB, Hyde C. An optimal variant to gene distance window derived from an empirical definition of cis and trans protein QTLs. BMC Bioinformatics. 2022;23: 169. doi:10.1186/s12859-022-04706-x

30. Dimas AS, Deutsch S, Stranger BE, Montgomery SB, Borel C, Attar-Cohen H, et al. Common regulatory variation impacts gene expression in a cell type-dependent manner. Science. 2009;325: 1246–50. doi:10.1126/science.1174148

31. Pfeifer D, Kist R, Dewar K, Devon K, Lander ES, Birren B, et al. Campomelic dysplasia translocation breakpoints are scattered over 1 Mb proximal to SOX9: evidence for an extended control region. Am J Hum Genet. 1999;65: 111–24. doi:10.1086/302455

32. Schwerk J, Savan R. Translating the untranslated region. J Immunol. 2015;195: 2963–71. doi:10.4049/jimmunol.1500756

33. Dixon AL, Liang L, Moffatt MF, Chen W, Heath S, Wong KCC, et al. A genome-wide association study of global gene expression. Nat Genet. 2007;39: 1202–7. doi:10.1038/ng2109

34. Gilad Y, Rifkin SA, Pritchard JK. Revealing the architecture of gene regulation: the promise of eQTL studies. Trends Genet. 2008;24: 408–15. doi:10.1016/j.tig.2008.06.001

35. Emilsson V, Thorleifsson G, Zhang B, Leonardson AS, Zink F, Zhu J, et al. Genetics of gene expression and its effect on disease. Nature. 2008;452: 423–8.

doi:10.1038/nature06758

36. Hu Y, Li M, Lu Q, Weng H, Wang J, Zekavat SM, et al. A statistical framework for cross-tissue transcriptome-wide association analysis. Nat Genet. 2019;51: 568–576. doi:10.1038/s41588-019-0345-7

37. Fontanesi L, Schiavo G, Galimberti G, Calò DG, Scotti E, Martelli PL, et al. A genome wide association study for backfat thickness in Italian Large White pigs highlights new regions affecting fat deposition including neuronal genes. BMC Genomics. 2012;13: 583. doi:10.1186/1471-2164-13-583

38. Cheng F, Liang J, Yang L, Lan G, Wang L, Wang L. Systematic identification and comparison of the expressed profiles of lncRNAs, miRNAs, circRNAs, and mRNAs with associated co-expression networks in pigs with low and high intramuscular fat. Animals. 2021;11: 3212. doi:10.3390/ani11113212

39. Tao X, Liang Y, Yang X, Pang J, Zhong Z, Chen X, et al. Transcriptomic profiling in muscle and adipose tissue identifies genes related to growth and lipid deposition. PLoS One. 2017;12: e0184120. doi:10.1371/journal.pone.0184120

40. Bovo S, Mazzoni G, Bertolini F, Schiavo G, Galimberti G, Gallo M, et al. Genome-wide association studies for 30 haematological and blood clinical-biochemical traits in Large White pigs reveal genomic regions affecting intermediate phenotypes. Sci Rep. 2019;9: 7003. doi:10.1038/s41598-019-43297-1

41. Huang T, Jiang C, Yang M, Xiao H, Huang X, Wu L, et al. Salmonella enterica serovar Typhimurium inhibits the innate immune response and promotes apoptosis in a ribosomal/TRP53-dependent manner in swine neutrophils. Vet Res. 2020;51: 105. doi:10.1186/s13567-020-00828-3

42. Lunney JK, Ho C-S, Wysocki M, Smith DM. Molecular genetics of the swine major histocompatibility complex, the SLA complex. Dev Comp Immunol. 2009;33: 362–

74. doi:10.1016/j.dci.2008.07.002

43. Hublitz P, Kunowska N, Mayer UP, Müller JM, Heyne K, Yin N, et al. NIR is a novel INHAT repressor that modulates the transcriptional activity of p53. Genes Dev. 2005;19: 2912–24. doi:10.1101/gad.351205

44. Ivanov A V., Peng H, Yurchenko V, Yap KL, Negorev DG, Schultz DC, et al. PHD domain-mediated E3 ligase activity directs intramolecular sumoylation of an adjacent bromodomain required for gene silencing. Mol Cell. 2007;28: 823–37. doi:10.1016/j.molcel.2007.11.012

45. Liang Q, Deng H, Li X, Wu X, Tang Q, Chang T-H, et al. Tripartite motif-containing protein 28 is a small ubiquitin-related modifier E3 ligase and negative regulator of IFN regulatory factor 7. J Immunol. 2011;187: 4754–63. doi:10.4049/jimmunol.1101704

46. Chang J, Hwang HJ, Kim B, Choi Y-G, Park J, Park Y, et al. TRIM28 functions as a negative regulator of aggresome formation. Autophagy. Taylor & Francis; 2021;17: 4231–4248. doi:10.1080/15548627.2021.1909835

47. Zhai Y, Zhang M, An X, Zhang S, Kong X, Li Q, et al. TRIM28 maintains genome imprints and regulates development of porcine SCNT embryos. Reproduction. 2021;161: 411–424. doi:10.1530/REP-20-0602

48. Michalczyk AA, Ackland ML. hZip1 (hSLC39A1) regulates zinc homoeostasis in gut epithelial cells. Genes Nutr. 2013;8: 475–86. doi:10.1007/s12263-013-0332-z

49. Manning BJ, Yusufzai T. The ATP-dependent chromatin remodeling enzymes CHD6, CHD7, and CHD8 exhibit distinct nucleosome binding and remodeling activities. J Biol Chem. 2017;292: 11927–11936. doi:10.1074/jbc.M117.779470

50. Uhlen M, Zhang C, Lee S, Sjöstedt E, Fagerberg L, Bidkhori G, et al. A pathology atlas of the human cancer transcriptome. Science. 2017;357: eaan2507.

doi:10.1126/science.aan2507

51. Gondret F, Vincent A, Houée-Bigot M, Siegel A, Lagarrigue S, Causeur D, et al. A transcriptome multi-tissue analysis identifies biological pathways and genes associated with variations in feed efficiency of growing pigs. BMC Genomics. 2017;18: 244. doi:10.1186/s12864-017-3639-0

52. Fernández-Barroso MÁ, Caraballo C, Silió L, Rodríguez C, Nuñez Y, Sánchez-Esquiliche F, et al. Differences in the loin tenderness of Iberian pigs explained through dissimilarities in their transcriptome expression profile. Animals. 2020;10: 1715. doi:10.3390/ani10091715

53. Gurtner A, Manni I, Fuschi P, Mantovani R, Guadagni F, Sacchi A, et al. Requirement for down-regulation of the CCAAT-binding activity of the NF-Y transcription factor during skeletal muscle differentiation. Mol Biol Cell. 2003;14: 2706–15. doi:10.1091/mbc.e02-09-0600

54. Lee J, Kang J-H, Kim J-M. Bayes factor-based regulatory gene network analysis of genome-wide association study of economic traits in a purebred swine population. Genes (Basel). 2019;10: 293. doi:10.3390/genes10040293

55. Sharma S, Sourirajan A, Baumler DJ, Dev K. Saccharomyces cerevisiae ER membrane protein complex subunit 4 (EMC4) plays a crucial role in eIF2B-mediated translation regulation and survival under stress conditions. J Genet Eng Biotechnol. Journal of Genetic Engineering and Biotechnology; 2020;18: 15. doi:10.1186/s43141-020-00029-7

56. Richard M, Boulin T, Robert VJP, Richmond JE, Bessereau J-L. Biosynthesis of ionotropic acetylcholine receptors requires the evolutionarily conserved ER membrane complex. Proc Natl Acad Sci U S A. 2013;110: E1055-63. doi:10.1073/pnas.1216154110

57. Fan H, Chu J-Y. A brief review of short tandem repeat mutation. Genomics Proteomics Bioinformatics. Beijing Institute of Genomics; 2007;5: 7–14. doi:10.1016/S1672-0229(07)60009-6

58. Yang Q, Doublié S. Structural biology of poly(A) site definition. Wiley Interdiscip Rev RNA. 2011;2: 732–47. doi:10.1002/wrna.88

59. Krasilnikov AS, Mondragón A. On the occurrence of the T-loop RNA folding motif in large RNA molecules. RNA. 2003;9: 640–3. doi:10.1261/rna.2202703

60. Iyer V, Struhl K. Poly(dA:dT), a ubiquitous promoter element that stimulates transcription via its intrinsic DNA structure. EMBO J. 1995;14: 2570–9. doi:10.1002/j.1460-2075.1995.tb07255.x

61. Segal E, Widom J. Poly(dA:dT) tracts: major determinants of nucleosome organization. Curr Opin Struct Biol. 2009;19: 65–71. doi:10.1016/j.sbi.2009.01.004

62. INRAE UE3P France Génétique Porc phenotyping facilities. doi:10.15454/1.5573932732039927E12

63. Acloque H, Mongellaz M. Purification of Genomic DNA and RNA, from liver and skeletal muscle of adult pigs. 2021. Available: https://data.faang.org/api/fire_api/experiments/INRAE_SOP_GENESWITCH_WP4_EXTRACTION_DNA_RNA_20210630.pdf

64. González-Rodríguez O, Ballester M. Purification of Genomic DNA from blood and RNA from liver, duodenum and skeletal muscle of adult pigs. 2022. Available: https://data.faang.org/api/fire_api/experiments/IRTA_SOP_EXTRACTION_DNA_RNA_20220725.pdf

65. Andrews S. FastQC: a quality control tool for high throughput sequence data. 2010. Available: http://www.bioinformatics.babraham.ac.uk/projects/fastqc

66. Li H. Aligning sequence reads, clone sequences and assembly contigs with BWA-MEM. arXiv. 2013; Available: https://arxiv.org/abs/1303.3997

67. McKenna A, Hanna M, Banks E, Sivachenko A, Cibulskis K, Kernytsky A, et al. The genome analysis toolkit: A MapReduce framework for analyzing next-generation DNA sequencing data. Genome Res. 2010;20: 1297–1303. doi:10.1101/gr.107524.110

68. Danecek P, Bonfield JK, Liddle J, Marshall J, Ohan V, Pollard MO, et al. Twelve years of SAMtools and BCFtools. Gigascience. 2021;10: giab008. doi:10.1093/gigascience/giab008

69. Chang CC, Chow CC, Tellier LC, Vattikuti S, Purcell SM, Lee JJ. Second-generation PLINK: rising to the challenge of larger and richer datasets. Gigascience. 2015;4: 7. doi:10.1186/s13742-015-0047-8

70. Dobin A, Davis CA, Schlesinger F, Drenkow J, Zaleski C, Jha S, et al. STAR: ultrafast universal RNA-seq aligner. Bioinformatics. 2013;29: 15–21. doi:10.1093/bioinformatics/bts635

71. Li B, Dewey CN. RSEM: accurate transcript quantification from RNA-Seq data with or without a reference genome. BMC Bioinformatics. 2011;12: 323. doi:10.1186/1471-2105-12-323

72. Robinson MD, McCarthy DJ, Smyth GK. edgeR: a Bioconductor package for differential expression analysis of digital gene expression data. Bioinformatics. 2010;26: 139–40. doi:10.1093/bioinformatics/btp616

73. Yang J, Lee SH, Goddard ME, Visscher PM. GCTA: a tool for genome-wide complex trait analysis. Am J Hum Genet. 2011;88: 76–82. doi:10.1016/j.ajhg.2010.11.011

74. Yang J, Benyamin B, McEvoy BP, Gordon S, Henders AK, Nyholt DR, et al.

Common SNPs explain a large proportion of the heritability for human height. Nat Genet. 2010;42: 565–9. doi:10.1038/ng.608

75. Kinsella RJ, Kähäri A, Haider S, Zamora J, Proctor G, Spudich G, et al. Ensembl BioMarts: a hub for data retrieval across taxonomic space. Database. 2011;2011: bar030. doi:10.1093/database/bar030

76. McLaren W, Gil L, Hunt SE, Riat HS, Ritchie GRS, Thormann A, et al. The Ensembl Variant Effect Predictor. Genome Biol. Genome Biology; 2016;17: 122. doi:10.1186/s13059-016-0974-4

77. Hu H, Miao Y-R, Jia L-H, Yu Q-Y, Zhang Q, Guo A-Y. AnimalTFDB 3.0: a comprehensive resource for annotation and prediction of animal transcription factors. Nucleic Acids Res. 2019;47: D33–D38. doi:10.1093/nar/gky822

78. Bindea G, Mlecnik B, Hackl H, Charoentong P, Tosolini M, Kirilovsky A, et al. ClueGO: a Cytoscape plug-in to decipher functionally grouped gene ontology and pathway annotation networks. Bioinformatics. 2009;25: 1091–3. doi:10.1093/bioinformatics/btp101

79. Shannon P, Markiel A, Ozier O, Baliga NS, Wang JT, Ramage D, et al. Cytoscape: a software environment for integrated models of biomolecular interaction networks. Genome Res. 2003;13: 2498–504. doi:10.1101/gr.1239303

80. Reverter A, Chan EKF. Combining partial correlation and an information theory approach to the reversed engineering of gene co-expression networks. Bioinformatics. 2008;24: 2491–2497. doi:10.1093/bioinformatics/btn482

81. Bailey TL, Johnson J, Grant CE, Noble WS. The MEME Suite. Nucleic Acids Res. 2015;43: W39-49. doi:10.1093/nar/gkv416

82. Bailey TL, Elkan C. Fitting a mixture model by expectation maximization to discover motifs in biopolymers. Proceedings Int Conf Intell Syst Mol Biol. 1994;2:

28–36. Available: http://www.ncbi.nlm.nih.gov/pubmed/7584402

83. Grant CE, Bailey TL, Noble WS. FIMO: scanning for occurrences of a given motif. Bioinformatics. 2011;27: 1017–1018. doi:10.1093/bioinformatics/btr064

84. Supporting Information: List of the 14,096,080 significant associations obtained by the eGWAS between the expressed genes of duodenum, liver and muscle tissues and polymorphisms distributed across the genome. Available: https://github.com/Daniel-Crespo/GENE-SWitCH/raw/main/TS1_eGWAS_results.tsv.gz

85. Crespo-Piazuelo D, Acloque H, González-Rodríguez O, Mongellaz M, Mercat MJ, Bink MCAM, et al. Supporting data for "Identification of transcriptional regulatory variants in pig duodenum, liver and muscle tissues". GigaScience Database. 2023. http://dx.doi.org/10.5524/102388

Figure1 Click here to access/download;Figure;Figure1.pdf

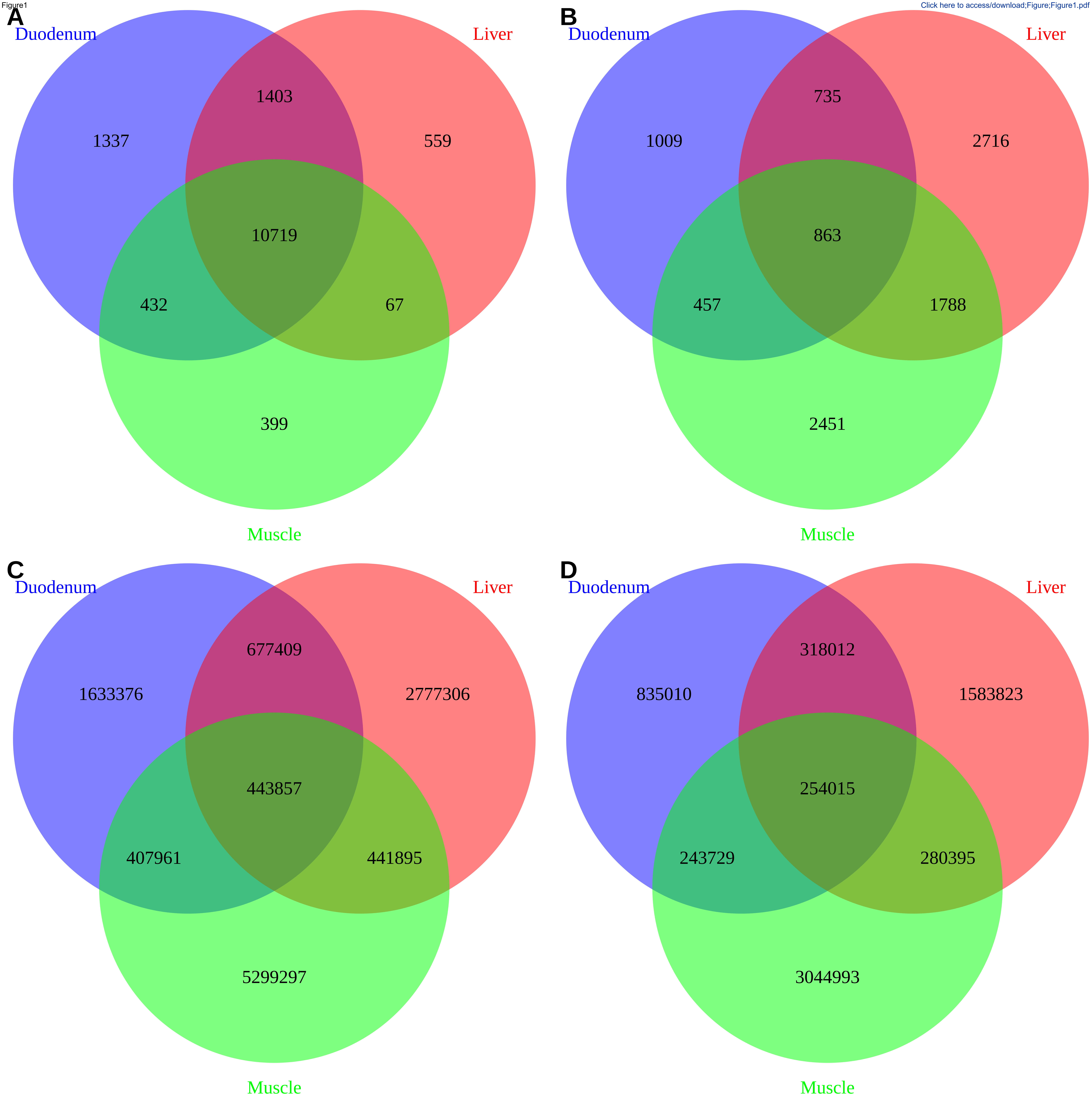

Figure2

Click here to access/download;Figure;Figure2.tiff

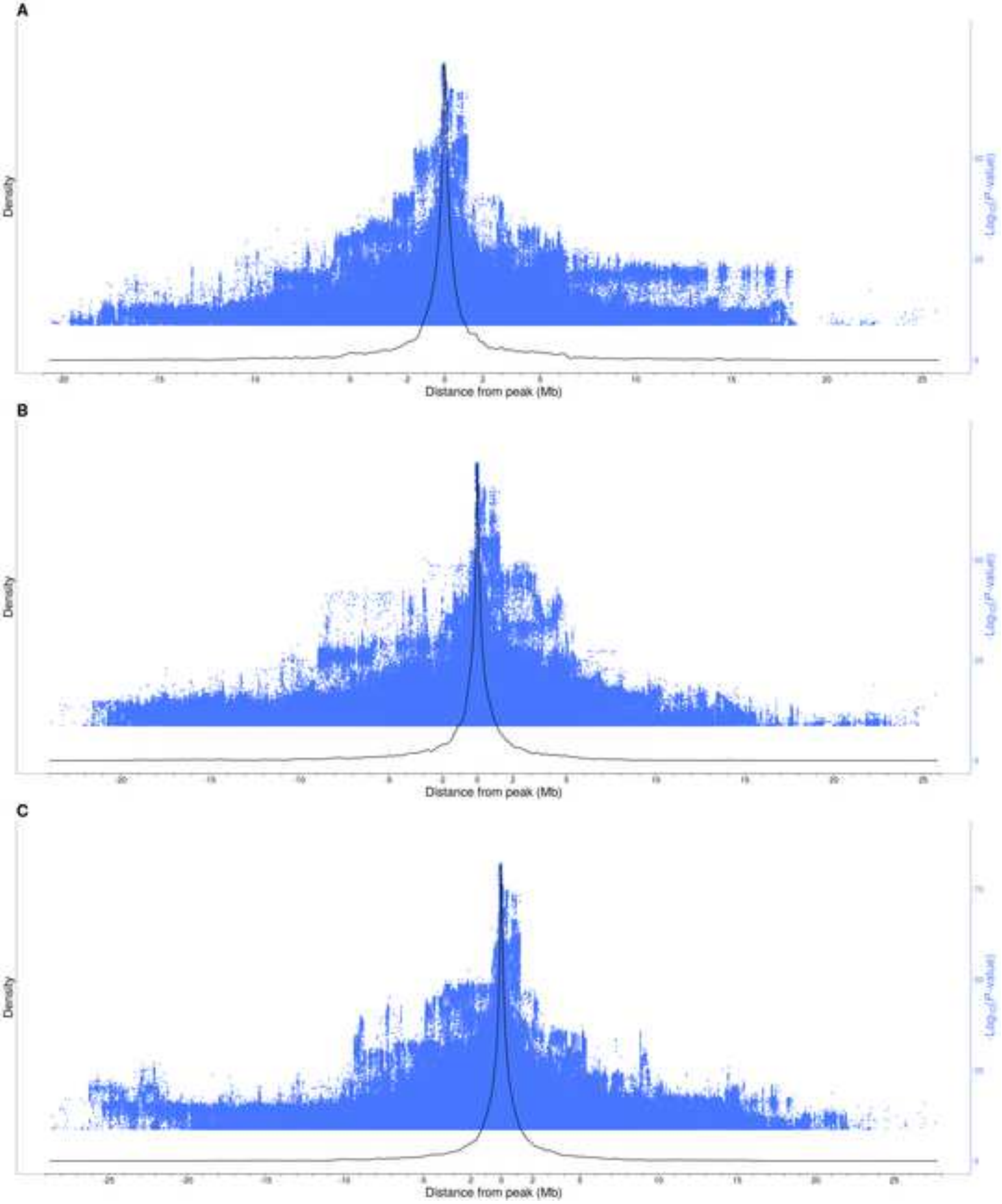

Figure3

Click here to access/download;Figure;Figure3.pdf

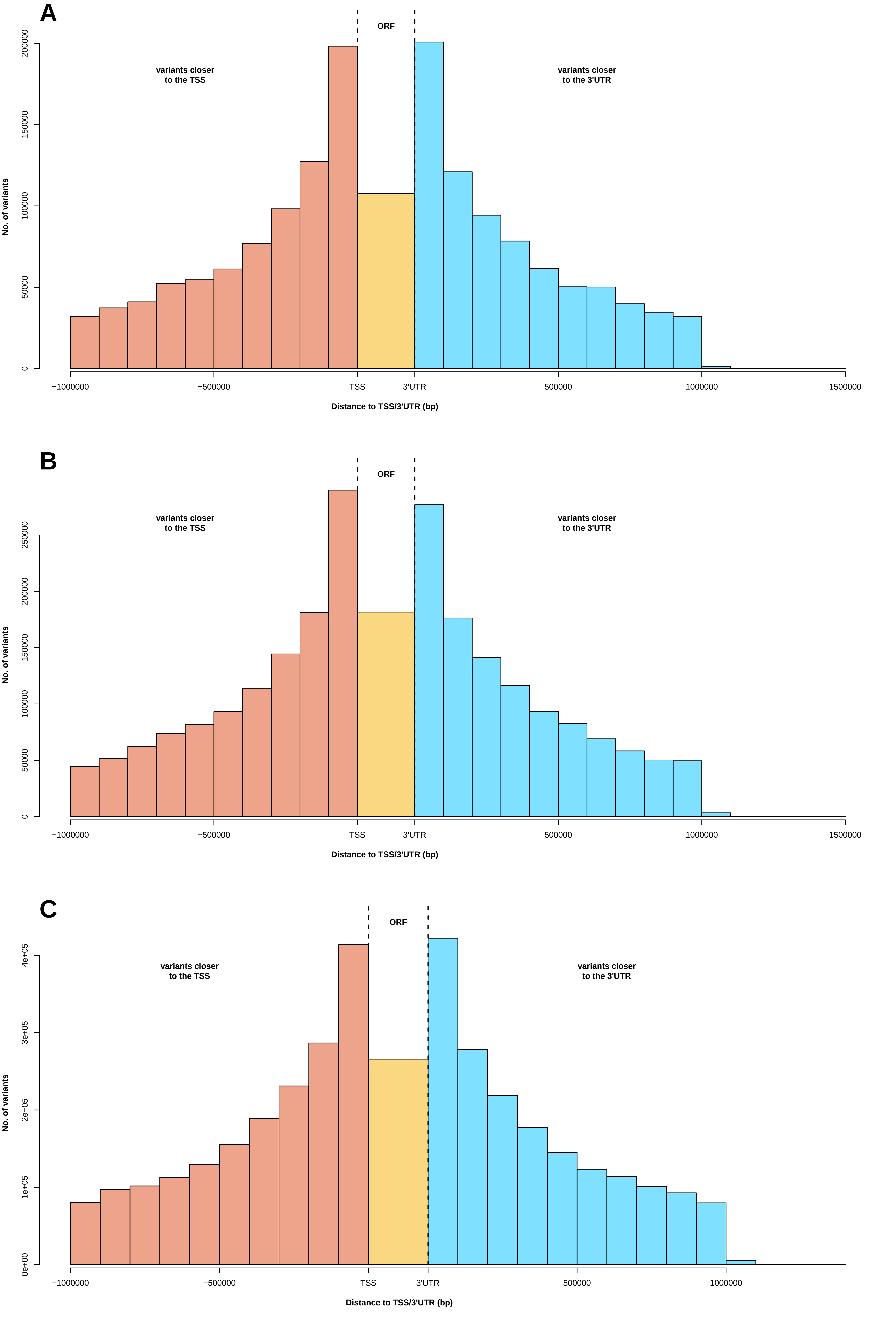

Figure4

Click here to access/download;Figure;Figure4.pdf

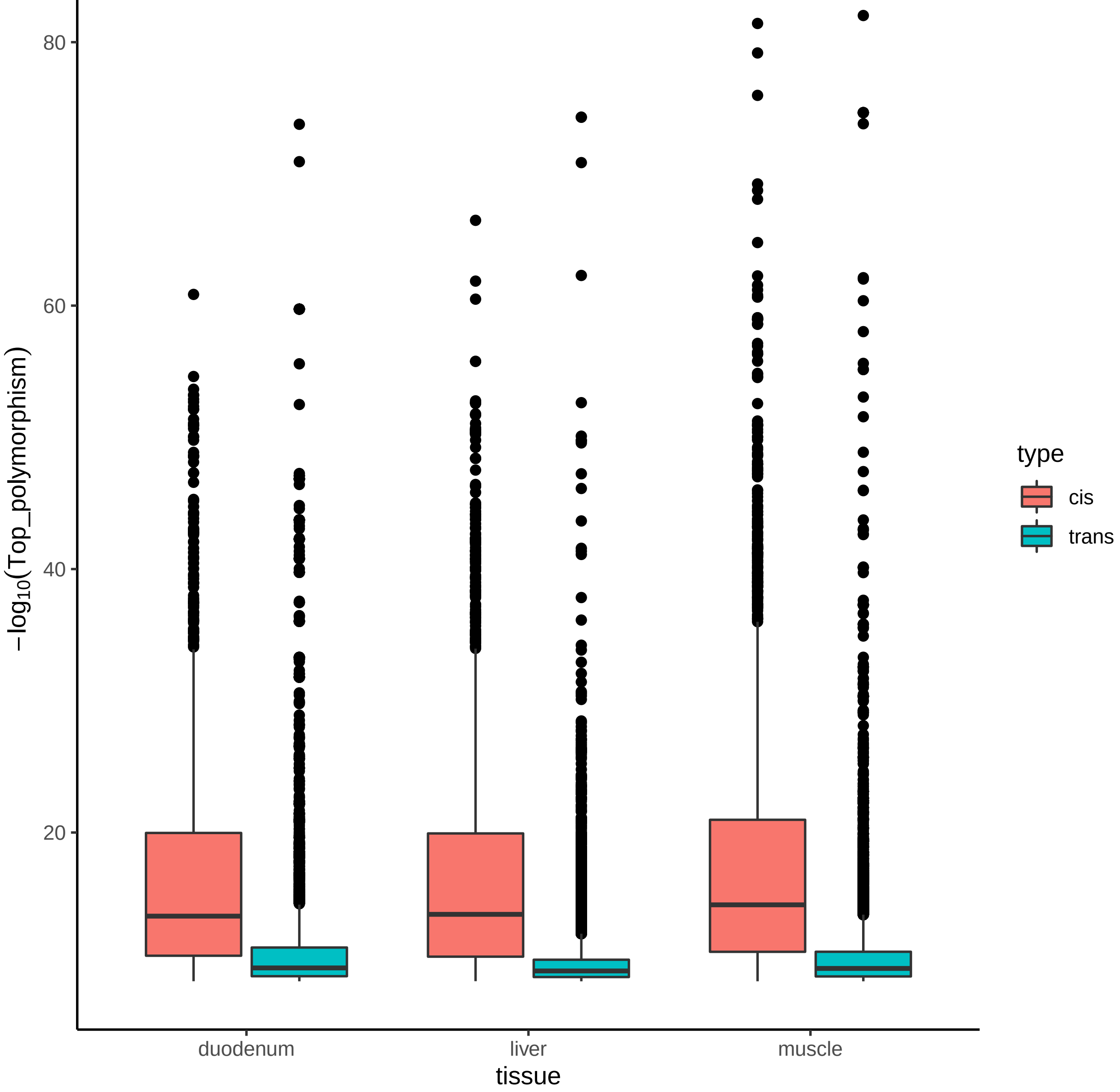

Figure5

Click here to access/download;Figure;Figure5.pdf

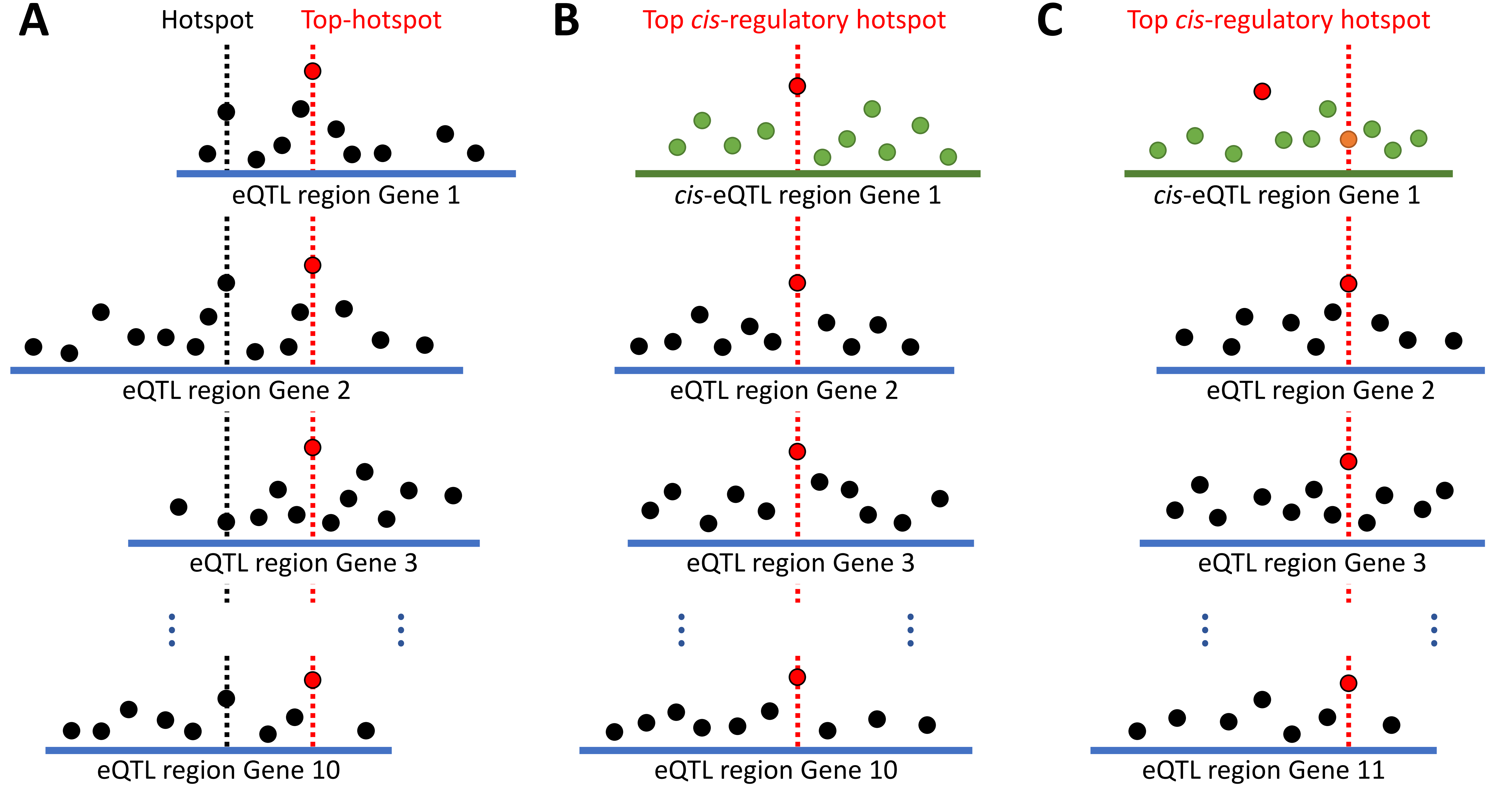

Click here to access/download;Figure;Figure6.png

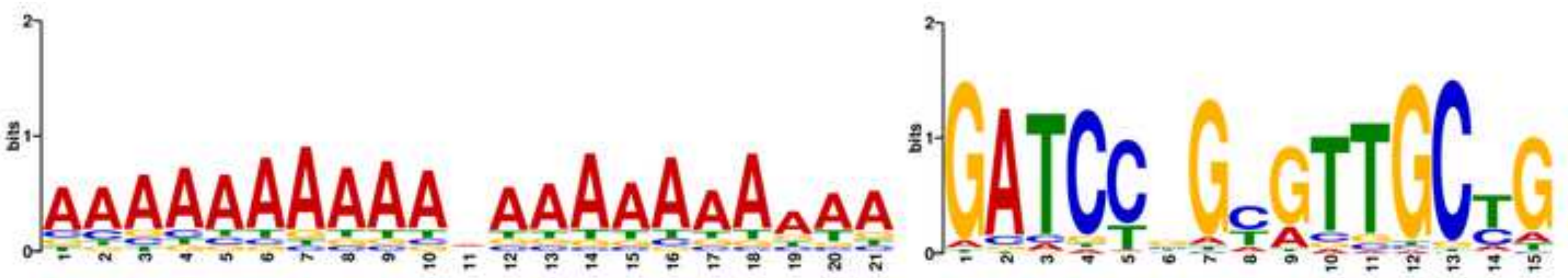

Supplementary_Table_S1

Click here to access/download
Supplementary Material
Supplementary_Table_S1.tsv

Supplementary_Table_S2

Click here to access/download
Supplementary Material
Supplementary_Table_S2.tsv

Click here to access/download
Supplementary Material
Supplementary_Table_S3.tsv

Click here to access/download
Supplementary Material
Supplementary_Table_S4.tsv

Click here to access/download
Supplementary Material
Supplementary_Table_S5.tsv

Cover Letter
Click here to access/download;Personal Cover;Cover_Letter.docx

Daniel Crespo Piazuelo
Animal Breeding and Genetics Program, IRTA, Torre Marimon, E08140
Caldes de Montbui, Spain

April 13th, 2023

Dear Editor,

Thank you very much for your e-mail of February 3rd concerning the manuscript GIGA-D-22-00301 now entitled “Identification of transcriptional regulatory variants in pig duodenum, liver and muscle tissues” which we submitted as a Research article to *GigaScience*.

We thank you and the reviewers for the time and expertise invested in reviewing our manuscript. We really appreciate the comments and suggestions and we truly believe that have helped to improve the manuscript. Following their recommendations, we have also improved the English and the readability of our manuscript.

We hope that the manuscript can be considered now acceptable for *GigaScience*.

Yours faithfully,

Daniel Crespo Piazuelo
on behalf of all co-authors
