# Supplementary material for: Identification of transcriptional regulatory variants in pig duodenum, liver, and muscle tissues
Source: Gigascience. 2023 Jun 24;12:giad042. doi: 10.1093/gigascience/giad042 (PMC10290502; doi:10.1093/gigascience/giad042)
Supplement: giad042_Response_to_Reviewer_Comments_Original_Submission [file giad042_response_to_reviewer_comments_original_submission.pdf]

Author's Response To Reviewer Comments

Close

We want to thank the reviewers for their insight and suggestions. Their feedback has been really helpful in improving the manuscript. Below, each commentary has been addressed separately.

Reviewer reports:
Reviewer #1: The manuscript of Crespo-Piazuelo et al. describes eQTL discovery in pigs by merging WGS and RNA-seq data. The authors carried out WGS in about 300 pigs from three populations (LW, DU and LA) and the characterization of the transcriptome from three different tissues (duodenum, liver and muscle). After the detection of associated variants, followed their evaluation as cis- or trans-regulatory elements as well as hotspots elements; genes associated with the same top-hotspot underwent to over-representation analyses (GO terms and KEGG pathways) and co-expression analyses of trans-associated-genes. Overall, the manuscript presents a proper introduction, interesting results and a proper discussion. Protocols, data and results have been properly released through the FAANG data portal. The manuscript can be accepted in the present from. I have only some minors related to the material and methods part:

- Line 114-115. To evaluate the goodness of the obtained dataset, it would be nice to report the Ts/Tv value.
The Ts/Tv value of the dataset was 2.01, which is good overall. The value has been included in the revised version of the manuscript (L117).

- Line 466. Please, provide info about the sex of animals and their relatedness.
Duroc animals were distributed in three batches balancing gender, 50 males and 50 females, and belonged to 33 litters obtained from 33 sows and 10 boars. Large-White pigs were distributed in 4 batches of uncastrated males and belonged to 84 litters obtained from 84 sows and 43 boars. Landrace animals were taken from one batch, 39 males and 61 females, and belonged to 74 litters obtained from 74 sows and 18 boars. This information has been added in the final version of the manuscript (L467-472).

- Line 492-499. It is not clear how many Gbp have been produced for each WGS and RNA-seq data. "Whole genome (>30Gb; n=300)" means that each genome was covered by at least 30Gbp resulting in a depth of sequencing of about 10X?
All DNA samples were sequenced with a yield > 30 Gb, resulting in a whole genome sequencing (WGS) depth of about 10X, the mean yield for all samples was 45.122 Gb with a standard deviation of 9.72.
For RNA-seq, all samples were sequenced with a minimum depth of 45 M PE reads (2x150 bp) per sample (>90M reads). The mean yield for muscle RNA samples was 23.851 Gb with a standard deviation of 7.48; for liver samples was 22.631 Gb with a standard deviation of 5.82 and 22.00 Gb with a standard deviation of 5.73 for duodenum samples.
We have modified the sentence to clarify the sequencing depth for each WGS and RNA-seq data (L499-501).

- Line 494. Insert size of the library is missing.
Whole genome sequencing and transcriptomic libraries were constructed with 470 bp and 285 bp insert sizes, respectively. These values have been included in the final version of the manuscript (L497-499).

- Line 503-507. Did you use HaplotypeCaller default parameters?
The only exception that we applied to the default parameters was increasing the minimum-mapping-quality from 10 to 20. This has been clarified on the manuscript (L512-513).

After calling SNPs/INDELs, GATK best practices suggest some filtering recommendations. Did you make use of any variant recalibration or hard-filtering?
First, bcftools norm was used to split multiallelic sites (SNPs and indels) into multiple rows. Then, variants

were flagged following GATK best practices for hard-filtering with the VariantFiltration tool. However, despite all the hard-filtering applied, at the end, only 0.8% out of the 25 million variants that passed the --maf 0.05 and --geno 0.1 PLINK filters were flagged variants. Thus, we considered that these flagged variants could be relevant as they were segregating in our population, and as such, we did not include GATK hard-filtering in the manuscript.

Did you retain only bi-allelic variants? Is the 5% used to retain a variant within breed or at the population level (in the second case, you are losing some variants).
We did not exclusively retain bi-allelic variants. We kept tri-allelic polymorphisms or variants such as microsatellites, albeit per notation and for the eGWAS, they were included and analysed as different variants against the reference on the same position. About 10.5% of the 25M polymorphisms shared the same position.
The reviewer is correct on the assertion that some variants were lost because of the 5% applied at population level for retaining a variant. However, we indeed tried the 5% filter at breed level on our first iteration, which is translated as 1.67% at population level, but this caused the eGWAS to be more prone to population structure artifacts. Therefore, as we were more focused on the common elements between breeds, we decided on applying the filtering at population level.

- Line 527. The GWAS model considers, for each tissue, the breed as covariate. What is the choice of this approach instead of running breed-specific GWASs followed by a meta-GWAS? Moreover, it would be nice to have, as results for the associated variants, the allele frequencies stratified by breed (not only at the level of the whole population).
The decision on analysing the samples in this fashion followed two main points. Speed was one of the main factors that moved us to analyse such data in such way. Performing each eGWAS per breed would have required thrice as much power computing and storage that with a merged dataset. In addition, it would have compromised the comprehension of the manuscript, as it would have required the discussion of each set in particular. Following this idea is the other main point, as we wanted to exclusively focus on those elements that were present in all the three breeds, that is why we chose to remove those genes that were missing in more than 20% of the animals. Nonetheless, we are considering the publication of a follow-up article that will analyse each breed individually using multi-tissue networks.

Reviewer #2: Crespo-Piazuelo et al collected duodenum, liver, and muscle tissues of 300 pigs from three different breeds (Duroc, Landrace and Large White), and performed whole genome and transcriptome sequencing to obtain eQTL regions. This is a huge project in pig breeding field. The study is very interesting especially the analysis of hotspot regulatory sits. However, I have several concerns for this manuscript.

1. The authors should integrate the GWAS or QTL data of economic phenotypes of pigs to improve the level of this manuscript.
We concur with the reviewer's opinion that the inclusion of economically important traits to the manuscript would be of importance for the field. However, this study, as part of the GENE-SWitCH European project, was more focused on studying the transcriptional regulatory elements of the genomes, rather than determining their relationship to few specific complex traits. In addition, the few overlapped recorded traits for the 300 animals are being used on other manuscript of our partners of the GENE-SWitCH European project to evaluate genomic predictions with the inclusion of transcriptomic data. Altogether, we are limited on the inclusion of such measurements on this manuscript.

2. Taking "Identification of transcriptional regulatory variants in pig duodenum, liver and muscle tissues" as paper title might be better.
Thank you for the suggestion, we agree with the reviewer and have amended the title as recommended.

3. Promoters, enhancers et al. are "cis-regulatory elements". It's better to change cis-regulatory elements to cis-regulatory SNPs or variations.
It is true that the word "cis-regulatory elements" may refer to any sort of loci or regions with a regulatory role over the gene. Hence, to avoid any misleading, we have used the term "cis-regulatory variants" instead as suggested.

4. The analysis of regulatory elements should incorporate the data sets of ChIP-seq and/or STARR-seq.
These two datasets have been generated by different partners of the GENE-SWitCH European project. Likewise, the manuscripts with this information will be made publicly available in other publication(s). In addition, the animals used to generate both datasets (ChIP-seq and STARR-seq) were different from those used in this study, differing in age and breed (only Durocs were used). Thus, we are unable to incorporate this information to the manuscript.

5. The English writing of the manuscript is of a little poor quality. The manuscript should be carefully proofread to improve the quality of writing.
Following the reviewer suggestion, the manuscript was proofread by a native English speaker, our colleague Dr. Andrea Rau. This has been reflected in the acknowledgements section.

Close
