# Supplementary material for: Identification of transcriptional regulatory variants in pig duodenum, liver, and muscle tissues
Source: Gigascience. 2023 Jun 24;12:giad042. doi: 10.1093/gigascience/giad042 (PMC10290502; doi:10.1093/gigascience/giad042)
Supplement: giad042_Reviewer_1_Report_Original_Submission — Samuele Bovo, Ph.D -- 1/24/2023 [file giad042_reviewer_1_report_original_submission.pdf]

Reviewer Report

Title: Identification of transcriptional regulatory variants in pig duodenum, liver and muscle tissues

Version: Original Submission **Date:** 1/24/2023

Reviewer name: Samuele Bovo, Ph.D.

Reviewer Comments to Author:

The manuscript of Crespo-Piazuelo et al. describes eQTL discovery in pigs by merging WGS and RNA-seq data. The authors carried out WGS in about 300 pigs from three populations (LW, DU and LA) and the characterization of the transcriptome from three different tissues (duodenum, liver and muscle). After the detection of associated variants, followed their evaluation as cis- or trans-regulatory elements as well as hotspots elements; genes associated with the same top-hotspot underwent to over-representation analyses (GO terms and KEGG pathways) and co-expression analyses of trans-associated-genes. Overall, the manuscript presents a proper introduction, interesting results and a proper discussion. Protocols, data and results have been properly released through the FAANG data portal. The manuscript can be accepted in the present from. I have only some minors related to the material and methods part:
- Line 114-115. To evaluate the goodness of the obtained dataset, it would be nice to report the Ts/Tv value.
- Line 466. Please, provide info about the sex of animals and their relatedness.
- Line 492-499. It is not clear how many Gbp have been produced for each WGS and RNA-seq data. "Whole genome (>30Gb; n=300)" means that each genome was covered by at least 30Gbp resulting in a depth of sequencing of about 10X?
- Line 494. Insert size of the library is missing.
- Line 503-507. Did you use HaplotypeCaller default parameters? After calling SNPs/INDELs, GATK best practices suggest some filtering recommendations. Did you make use of any variant recalibration or hard-filtering? Did you retain only bi-allelic variants? Is the 5% used to retain a variant within breed or at the population level (in the second case, you are losing some variants).
- Line 527. The GWAS model considers, for each tissue, the breed as covariate. What is the choice of this approach instead of running breed-specific GWASs followed by a meta-GWAS? Moreover, it would be nice to have, as results for the associated variants, the allele frequencies stratified by breed (not only at the level of the whole population).

Methods

Are the methods appropriate to the aims of the study, are they well described, and are necessary controls included? Choose an item.

Conclusions

Are the conclusions adequately supported by the data shown? Choose an item.

Reporting Standards

Does the manuscript adhere to the journal's guidelines on minimum standards of reporting? Choose an item.

Choose an item.

Statistics

Are you able to assess all statistics in the manuscript, including the appropriateness of statistical tests used? Choose an item.

Quality of Written English

Please indicate the quality of language in the manuscript: Choose an item.

Declaration of Competing Interests

Please complete a declaration of competing interests, considering the following questions:

- Have you in the past five years received reimbursements, fees, funding, or salary from an organisation that may in any way gain or lose financially from the publication of this manuscript, either now or in the future?
- Do you hold any stocks or shares in an organisation that may in any way gain or lose financially from the publication of this manuscript, either now or in the future?
- Do you hold or are you currently applying for any patents relating to the content of the manuscript?
- Have you received reimbursements, fees, funding, or salary from an organization that holds or has applied for patents relating to the content of the manuscript?
- Do you have any other financial competing interests?
- Do you have any non-financial competing interests in relation to this paper?

If you can answer no to all of the above, write 'I declare that I have no competing interests' below. If your reply is yes to any, please give details below.

I declare that I have NO competing interests.

I agree to the open peer review policy of the journal. I understand that my name will be included on my report to the authors and, if the manuscript is accepted for publication, my named report including any attachments I upload will be posted on the website along with the authors' responses. I agree for my report to be made available under an Open Access Creative Commons CC-BY license (http://creativecommons.org/licenses/by/4.0/). I understand that any comments which I do not wish to be included in my named report can be included as confidential comments to the editors, which will not be published.

Choose an item.

To further support our reviewers, we have joined with Publons, where you can gain additional credit to further highlight your hard work (see: https://publons.com/journal/530/gigascience). On publication of this paper, your review will be automatically added to Publons, you can then choose whether or not to claim your Publons credit. I understand this statement.

Yes Choose an item.
